# Evaluation of Multiple Methods for Quantification of Glycosaminoglycan Biomarkers in Newborn Dried Blood Spots from Patients with Severe and Attenuated Mucopolysaccharidosis-I

**DOI:** 10.3390/ijns6030069

**Published:** 2020-08-26

**Authors:** Zackary M. Herbst, Leslie Urdaneta, Terri Klein, Maria Fuller, Michael H. Gelb

**Affiliations:** 1 Department of Chemistry, University of Washington, Seattle, WA 98195, USA; zherbst@uw.edu; 2 National MPS Society, P.O. Box 14686, Durham, NC 27707-4686, USA; leslie@mpssociety.org (L.U.); terri@mpssociety.org (T.K.); 3 Genetics and Molecular Pathology, SA Pathology at Women’s and Children’s Hospital, North Adelaide 5006, Australia; maria.fuller@adelaide.edu.au; 4 School of Medicine, University of Adelaide, Adelaide 5005, Australia; 5 Department of Biochemistry, University of Washington, Seattle, WA 98195, USA

**Keywords:** newborn screening, glycosaminoglycans, mucopolysaccharidosis, hurler syndrome, scheie syndrome, mass spectrometry, biochemical genetics

## Abstract

All newborn screening (NBS) for mucopolysaccharidosis-I (MPS-I) is carried out by the measurement of α-iduronidase (IDUA) enzymatic activity in dried blood spots (DBS). The majority of low enzyme results are due to pseudodeficiencies, and studies from the Mayo Clinic have shown that the false positive rate can be greatly reduced by including a second-tier analysis of glycosaminoglycans (GAGs) in DBS as part of NBS. In the present study, we obtained newborn DBS from 13 patients with severe MPS-I and 2 with attenuated phenotypes. These samples were submitted to four different GAG mass spectrometry analyses in a comparative study: (1) internal disaccharide; (2) endogenous disaccharide; (3) Sensi-Pro; (4) Sensi-Pro Lite (a variation of Sensi-Pro with a simplified workflow). Patients with attenuated MPS-I show less GAG elevation than those with severe disease, and all MPS-I patients were separated from the reference range using all four methods. The minimal differential factor (lowest GAG marker level in MPS-I samples divided by highest level in the reference range of 30 random newborns) was about two for internal disaccharide, Sensi-Pro, and Sensi-Pro Lite methods. The endogenous disaccharide was clearly the best method with a minimal differential of 16-fold. This study supports use of second-tier GAG analysis of newborn DBS, especially the endogenous disaccharide method, as part of NBS to reduce the false positive rate.

## 1. Introduction

Worldwide newborn screening (NBS) of MPS-I (OMIM Entry #607014) is done exclusively by the first-tier measurement of α-iduronidase enzymatic activity (IDUA, EC 3.2.1.76) in dried blood spots (DBS), either by tandem mass spectrometry (MS/MS) or by digital microfluidics fluorometry [1,2]. The rate of below cutoff samples is about 20 per 100,000 newborns using MS/MS [1]. Many of the hits from MS/MS- and fluorometry-based enzyme analysis are false positives, because of the partial overlap of the reference and affected ranges [3] and because of the presence of pseudodeficiencies [4]. This is the typical situation with NBS for lysosomal storage diseases. It would be prudent to develop a second-tier test that could identify most of the false positives as part of NBS so as to minimize the number of follow-up cases and anxiety to families. As an example, MS/MS measurement of psychosine in DBS greatly reduces the number of false positives in NBS of Krabbe disease, as well as helping to stratify patients into early versus late-onset disease [5]. Biochemical measurement of psychosine is too time-consuming to be used for first-tier NBS, and it appears to be more powerful than DNA sequencing for prognosis [5].

IDUA is a lysosomal enzyme that is required for breakdown of the glycosaminoglycans (GAGs) heparan sulfate and dermatan sulfate in lysosomes, and there are numerous studies showing that these polymers are elevated in plasma and urine from MPS-I patients (shown later). GAG analysis in DBS is currently not used for first-tier NBS of MPS-I because the analysis time per sample is still too long, and the false positive rate is well above that seen with IDUA measurement [6,7]. However, there is a place for GAG measurement in DBS as part of NBS. Recent data shows that this measurement is a powerful tool for reducing false positives when carried out as a second stage NBS test after measurement of IDUA activity [8].

In the current study, we report data on the levels of GAG-derived biomarkers in newborn DBS from MPS-I patients using four different MS/MS approaches.

## 2. Methods

Studies with DBS were approved by the Univ. of Washington IRB board (number 5580, approved 7/1/2020). Staff at the National MPS Society used their registry to reach out to families with MPS-I patients for consent to participate in the study. In appropriate states, family members used published protocols to request a stored newborn DBS from their state NBS laboratory. De-identified information about the affected patient and the DBS was sent to the Gelb laboratory. Clinical information and genotype (when available) for all patients is provided as Table S1. The state-specific DBS storage conditions are also provided.

GAG analyses were carried out using standard operating procedures provided as Supplementary Material.

## 3. Results

### 3.1. GAG-Derived Biomarkers and Methods of Detection

**Figure 1:**
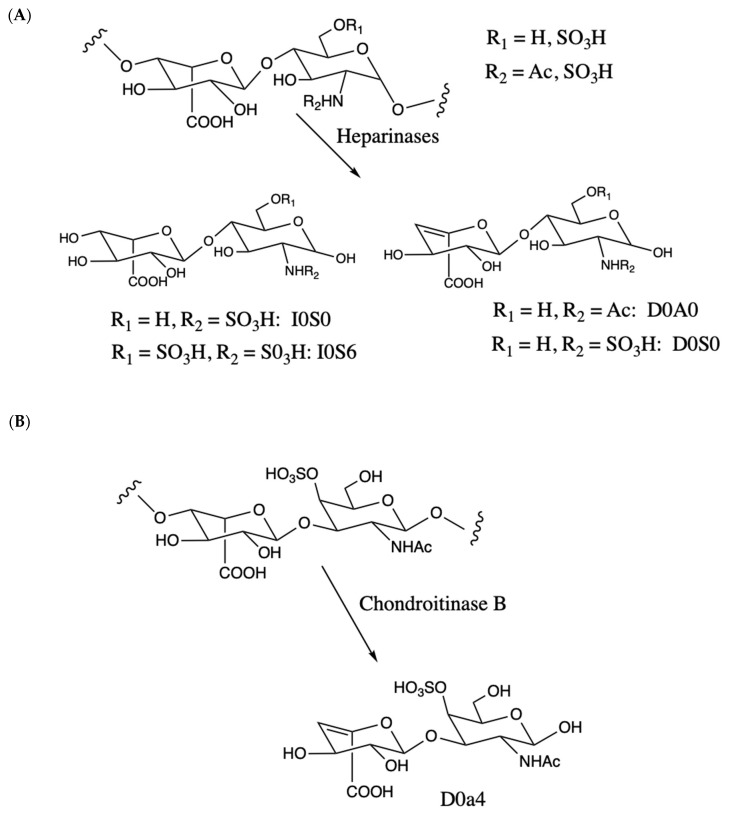
(**A**). Heparan sulfatide disaccharide repeat unit showing sulfation or acetylation on the amino group or sulfation on the 6-position of glucosamine. The 2-position of the iduronic acid residue can also be sulfated (not shown). Degradation with bacterial heparinase lyases yield internal disaccharides containing an unsaturated uronic acid (D0A0, D0S0, right) plus the non-reducing end disaccharides that lacks unsaturation (I0S0, I0S6, left). (**B**). Dermatan sulfate (chondroitin sulfate B) disaccharide repeat unit showing sulfation at the 4-position of GalNAc (major disaccharide in dermatan sulfate). Digestion with chondroitinase B lyase generates unsaturated internal disaccharides (D0a4) and the non-reducing end lacking unsaturation (not shown). In the internal disaccharide method, the disaccharides are analyzed without derivatization. With Sensi-Pro and Sensi-Pro Lite, the disaccharides are derived at carbon-1 of the amino-sugar with aniline/NaCNBH_3_ (not shown).

Iduronic acid is found in heparan sulfate and dermatan sulfate (also known as chondroitin sulfate B) GAG polymers. In MPS-I, deficiency of IDUA leads to accumulation of these polymers. By far, the most common approach to quantify these GAGs in DBS involves enzymatic degradation with bacterial lyases; heparinases for heparan sulfate and chondroitinase B for dermatan sulfate (Figure 1). Samples are treated with a mixture of three bacterial heparan sulfate lysases (heparinases I, II, and III from *Flavobacterium heparinum*). Each isoform has a specific preference for structural elements in the heparan sulfate chain, and the use of all three presumably results in more complete digestion of the GAG polymer to form disaccharides. Most of the previous studies measure the amounts of disaccharides that contain an unsaturated uronic acid as part of the heparinase- or chondroitinase-released disaccharide (Figure 1). Figure 1 also gives the names of the relevant disaccharides based on the conventions published previously [9]. Some laboratories use other abbreviated names, and a correspondence table is given as Table S2. We refer to this commonly used approach as the internal disaccharide method. Each GAG polymer yields many such disaccharide fragments as the GAG is a repeat of these units. LC-MS/MS is used to quantify the disaccharide fragments in underivatized form. Heparinase and chondroitinase B treatment also yields a single disaccharide, each of which lacks an unsaturated sugar (Figure 1), and this is called the non-reducing end because it is on the opposite end of the GAG chain from the end that is attached to the protein core of the proteoglycan (reducing end). The non-reducing end fragment is not analyzed in the internal disaccharide method.

Endogenous enzymes present in human tissues can also cleave GAG chains, and an endogenous disaccharide biomarker was reported to be elevated in urine from MPS-I patients [10]. This biomarker contains a uronic acid (iduronic acid or glucuronic acid) linked to N-acetyl-hexosamine (GlcNAc or GalNAc) and contains a single sulfate [11]. Its exact structure is unknown, and we refer to it as UA-HNAc(1S) (presumably UA is iduronic acid rather than glucuronic acid, but this was not proven). The endogenous enzyme(s) are hydrolases rather than lyases, as the biomarker lacks an unsaturated uronic acid. The biomarker presumably comes from the non-reducing end of the GAG polymer, but it could also be an internal disaccharide that is liberated by a double enzymatic cleavage of the chain.

The third method is Sensi-Pro [12]. It involves a combination of protease digestion, degradation of GAG polymers with heparinases (Figure 1), and derivatization of the reducing ends of the disaccharides. Both the internal disaccharides and non-reducing disaccharide can be quantified in the same LC-MS/MS analysis, but Sensi-Pro makes use of the non-reducing end only.

The GAG analysis methods as well as a modification of Sensi-Pro developed in the current study (Sensi-Pro Lite) are summarized in Table 1. The internal disaccharide method does not involve a derivatization step. Without derivatization, the analytes are highly polar and are analyzed using hydrophilic-interaction HPLC columns (HILIC-type). Internal standards used are structural analogs of the biomarkers (i.e., chondrosine). A disadvantage of this is that the internal standard does not accurately account for suppression of ionization of the analytes by matrix components since the internal standard and analyte do not co-elute during LC. Additionally, the MS/MS response factors for analytes and internal standard are different. See Discussion for a more complete analysis of this problem. In cases where the internal standard and analyte are not chemically identical, we report apparent analyte levels defined in Supplementary Material.

In the endogenous disaccharide method, the analytes are derivatized with 1-phenyl-3-methyl-5-pyrazolone (PMP). This adds a hydrophobic element to the structures so that LC can be carried out with conventional reverse-phase columns. Internal standards are not currently available for this method, and the structural analog chondroitin disaccharide di-4S (Carbosynth, Inc., Berkshire, UK) was used (efforts are underway in the Gelb laboratory to determine the precise structure of the marker and to prepare an internal standard). Sensi-Pro involves derivatization with aniline/NaCNBH_3_. This hydrophobic tagging permits use of reverse-phase LC columns. Another reason for use of aniline derivatization is that it allows for the preparation of a chemically-identical, but isotopically-distinguished, internal standard. This is prepared by derivatization of chemically synthesized non-reducing end disaccharide with deuterium labeled aniline [12].

### 3.2. Results Using the Internal Disaccharide Method

Several papers using this method have been published by Tomatsu and co-workers (for example, [13]), and our initial studies were carried out using the protocol reported by this laboratory. After incubation of the DBS punch in buffer with heparinases, the sample is submitted to ultra-filtration using a 96-well filter plate. We explored an alternative method of quenching the enzymatic digest with methanol to precipitate proteins followed by centrifugation to remove insolubles. As a first step, we showed that the internal disaccharides D0A0, D0S0, and D0a4 as well as chondrosine internal standard are soluble in 80% methanol. Solutions of authentic disaccharides in water (10–500 pmole in 50 μL) were treated with 200 μL methanol or with 200 μL water. After centrifugation (15 min at 3000× *g*), the supernatant was analyzed by LC-MS/MS. No differences were observed in the water versus methanol mixtures (not shown), thus, showing that all the disaccharides remain soluble in 80% methanol.

A set of three MPS-I patient DBS samples and a healthy DBS control were used to compare the filtration and methanol quenching methods side-by-side. After incubation of DBS punches with heparinases and chondroitinase B, a fraction of the mixture was removed (20 μL) and to this was added 80 μL of methanol. After centrifugation, the supernatant was removed and analyzed by LC-MS/MS. The remaining enzyme reaction mixture (~120 μL) was filtered through the filter plate and processed for LC-MSMS. Results indicate that the two methods yield equivalent results. Levels of D0A0, D0S0, and D0a4 markers were equally elevated in MPS-I samples compared to healthy DBS controls (not shown).

The bacterial heparinases are known to be Ca^2+^ dependent [14,15]. Enzymatic activity was completely abolished if EDTA was added to the buffer to remove enzyme-bound Ca^2+^. However, in the absence of chelating agent and added Ca^2+^, heparinase activity was about 50% of that measured after Ca^2+^ supplementation. Altogether, the data suggest that routine laboratory buffers contain sufficient Ca^2+^ as a contaminant in buffer reagents and water to support partial activity of the heparinases. In all previous reports of GAG-derived disaccharides reported by Tomatsu et al., heparinase digest buffer was not supplemented with Ca^2+^ (for example, [13]). We carried out a comparison study using the internal disaccharide method using buffer with and without added Ca^2+^. The results in Figure S1 show similar disaccharide levels indicating that Ca^2+^ addition is not necessarily essential. Nevertheless, in all subsequent studies we added Ca^2+^ to the heparinase digestion buffer to avoid possible fluctuation in results from variation in the amount of contaminating Ca^2+^ in non-supplemented buffers. Some laboratories also add dithiothreitol to the digestion buffer (personal communication with D. Matern, Mayo Clinic, Rochester, MN, USA), and we included this reductant in our buffer to minimize the chance of oxidative damage to the lyases.

**Figure 2:**
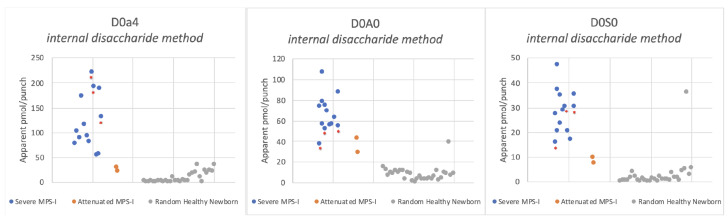
Levels of heparan sulfate-derived (D0A0, D0S0) and dermatan sulfate-derived (D0a4) internal disaccharides measured in dried blood spots (DBS) from newborn mucopolysaccharidosis-I (MPS-I) patients using the internal disaccharide method with heparinase digestion in buffer with Ca^2+^ and dithiothreitol. As a result of the limited sample, DBS from attenuated MPS-I patients were processed only in buffer without Ca^2+^ and dithiothreitol; however, data in Figure S1 suggest that GAG biomarker levels would not change significantly if Ca^2+^ and DTT were used. Note that a single random newborn gave high D0A0 and D0S0 levels. This same sample showed high D0A0 and D0S0 levels in the Sensi-Pro Lite analysis (Figure 7). Asterisked dots are from newborn DBS that were stored at ambient temperature, all others were stored frozen.

Figure 2 shows the levels of internal disaccharides derived from heparan sulfate (D0A0 and D0S0) and dermatan sulfate (D0a4) in newborn DBS from 13 severe MPS-I patients, 2 attenuated MPS-I patients, and 30 random newborns (presumably non-MPS-I). Apparent pmol of biomarker per DBS punch were obtained using chondrosine as an internal standard.

### 3.3. Results Using the Endogenous Disaccharide Method

As a first step toward establishing the endogenous disaccharide method [10] in our laboratory, we measured the MPS-I marker UA-HNAc(1S) as its PMP derivative in urine samples from all types of MPS patients (MPS-I, -II, -IIIA, -IIIB, -IIIC, -IIID, -IVA, -IVB, -VI, and VII) and found relatively high levels only in MPS-I urine (Table S3, consistent with the previous report [10]). We also studied fibroblasts from most types of MPS patients and found high level MPS-I marker only in MPS-I cells (Table S3). These results show the exquisite specificity of UA-HNAc(1S) for MPS-I as reported previously [10,11].

**Figure 3:**
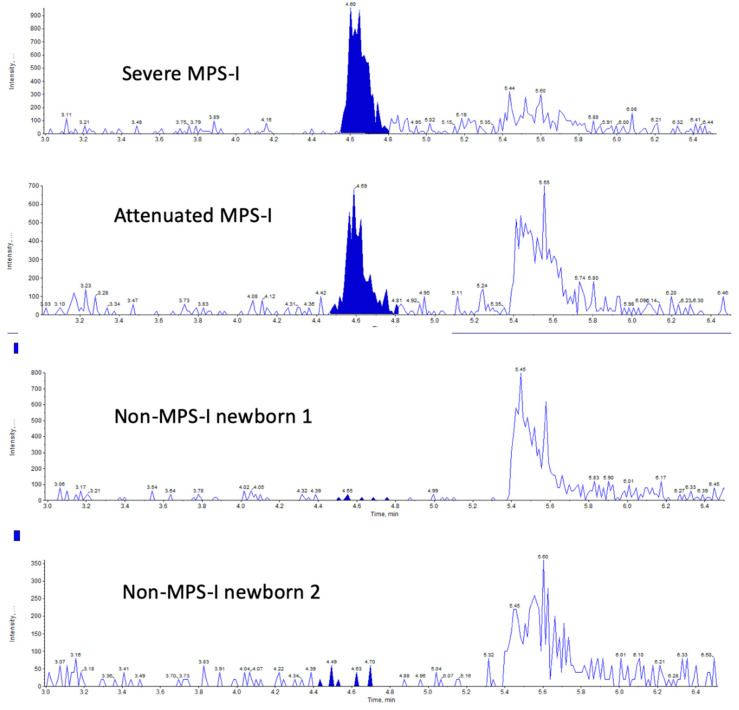
LC-tandem mass spectrometry (MS/MS) traces (multiple reaction monitoring (MRM) signal response versus time) for the endogenous disaccharide marker UA-HNAc(1S) 1-phenyl-3-methyl-5-pyrazolone (PMP) derivative using newborn DBSs from indicated patients.

**Figure 4:**
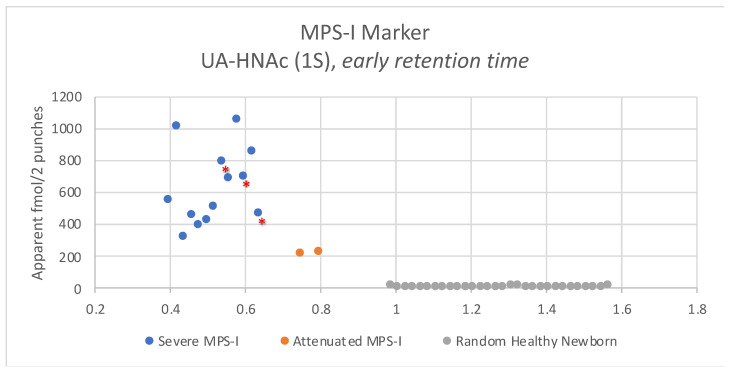
Levels of endogenous disaccharide biomarker UA-HNAc(1S) PMP derivative in newborn DBS from 30 random newborns (non-MPS-I), from 13 newborns with severe MPS-I, and from 2 newborns with attenuated MPS-I. Asterisked dots are from newborn DBS that were stored at ambient temperature, all others were stored frozen.

We then analyzed the MPS-I endogenous GAG marker in newborn DBS samples. Figure 3 shows typical LC-MS/MS chromatograms using two 3 mm punches using a newborn DBS from a severe MPS-I patient, an attenuated MPS-I patient, and two random newborns. Well defined multiple reaction monitoring (MRM) peaks were seen for the two MPS-I samples, but the signal for the two non-MPS-I samples was essentially noise-level. Figure 4 shows the level of the UA-HNAc(1S) marker as its PMP derivative measured using two 3 mm DBS punches from 30 random newborns (presumably non-MPS-I), from 13 newborns with severe MPS-I, and from two newborns with attenuated MPS-I. Apparent fmole per two 3 mm DBS punches is given based on use of PMP-derivatized chondroitin disaccharide di-4S as an internal standard.

In the same LC-MS/MS runs used to measure the MPS-I marker, we also included MRM channels for the other endogenous markers for each MPS sub-type (except MPS-VI, since we could not detect the MPS-VI endogenous biomarker in urine and fibroblasts from confirmed MPS-VI patients). Figure S2 shows the levels of MPS-IIIA, -IIIB, -IIIC, -IIID, -IVA, -IVB, and -VII biomarkers using newborn DBS from 30 random newborns, 13 severe MPS-I patients and two attenuated MPS-I patients. In all cases, the level of biomarkers in the MPS-I samples overlapped with the reference range (non-MPS-I samples) or slight elevations were seen for a subset of the MPS-I samples. As expected these additional biomarkers are overall not useful for detection of MPS-I patients.

**Figure 5:**
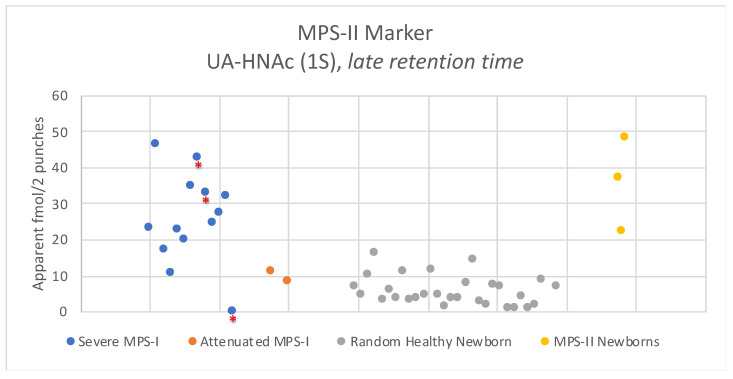
Levels of endogenous disaccharide biomarker UA-HNAc(1S) (late retention time) PMP derivative in newborn DBS from 30 random newborns (non-MPS-I), from 13 severe MPS-I patients, from two attenuated MPS-I patients, and from three MPS-II patients. Asterisked dots are from newborn DBS that were stored at ambient temperature, all others were stored frozen.

The exception was the endogenous biomarker for MPS-II. The MPS-II biomarker is designated UA-HNAc(1S)-late retention time, since it is isobaric with the MPS-I marker but elutes later in the LC run. Results are shown in Figure 5. Levels of this marker in newborn MPS-I DBS were above the reference range (30 random newborns) for most of the severe MPS-I samples. Three of 13 severe MPS-I DBS and both attenuated MPS-I DBS showed levels overlapping with the reference range. The elevation seen with some of the severe MPS-I DBS were comparable to the levels seen in newborn DBS from three MPS-II patients (Figure 5). The MPS-I newborn DBS showing the lowest amount of the MPS-II marker was one of the three samples stored at ambient temperature rather than frozen (Figure 5), suggesting that this marker is not stable without freezing. The MPS-II endogenous marker provides some diagnostic power for MPS-I, but is clearly not as reliable as the MPS-I biomarker (compare Figure 4 and Figure 5).

The endogenous disaccharide method uses a very large excess of PMP reagent to derivatize the biomarkers. When PMP is detected using a dedicated MRM channel, it is fount that it is fully eluted from the LC column before 3 min, and the 0–3 min region is diverted to waste rather than to the MS/MS ionization source. Thus, there is no concern with use of PMP.

### 3.4. Results with Sensi-Pro

The original Sensi-Pro method is a multi-step process [12]. The sample is first treated with the non-specific protease Pronase to digest the protein core of the proteoglycans. The highly anionic GAG polysaccharides are captured on an anion exchange resin, which is washed to elute non-bound material followed by washing with high salt buffer to elute the GAG chains. The latter are then desalted by passage through a column of size-exclusion gel. The GAG-containing fraction is concentrated to dryness then digested overnight with heparinases and dried again. The original Sensi-Pro method uses ammonium acetate buffer for heparinase digestion, which is followed by acylation of buffer ammonia using propionic anhydride [12]. We replaced the ammonium acetate buffer with HEPES to avoid the acylation step. The next step was derivatization in acetic acid/dimethylsulfoxide mixed solvent with aniline in the presence of NaCNBH_3_. This leads to attachment of aniline to carbon-1 of the reducing end of all GAG-derived fragments including the non-reducing end and the internal disaccharides. After solvent removal, the residue was taken up in solvent for injection onto LC-MS/MS.

We measured the level of the two Sensi-Pro markers I0S0 and I0S6 (Figure 1) in newborn DBS samples using internal standards that contained six deuteriums in the aniline tag (thus, chemically identical to the analytes). As shown in Figure S3, the signal for I0S6 was quite noisy, and it was difficult to obtain an accurate integral of the I0S6 peak. We obtained obtain more accurate values of I0S6 by using the MRM channel for I0S0. I0S0 and I0S6 have different LC retention times, and some of the I0S6 undergoes chemical cleavage to lose the sulfate group in the heated electrospray ionization source and, thus, appears in the MRM channel for I0S0 giving peaks, which can be accurately integrated (Figure S4).

**Figure 6:**
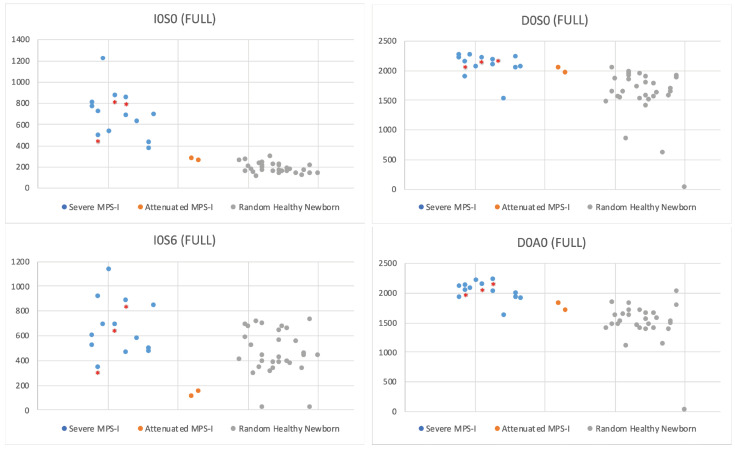
Sensi-Pro markers (I0S0 and I0S6) in DBS from 30 random newborns (non-MPS-I), 13 severe MPS-I newborns, and two attenuated MPS-I newborns. The level of internal disaccharide markers D0A0 and D0S0 measured in the same LC-MS/MS run are also shown. Asterisked dots are from newborn DBS that were stored at ambient temperature, all others were stored frozen.

Figure 6 shows the levels of Sensi-Pro biomarkers in newborn DBS from 13 severe MPS-I patients, two attenuated MPS-I patients, and 30 random newborns. The levels of internal disaccharide markers (D0A0 and D0S0) measured in the same LC-MS/MS run (these along with the non-reducing end fragments are produced by enzymatic digestion of GAGs) are also shown. Values for all samples are provided in Table S4.

### 3.5. Results with Sensi-Pro Lite

Given the complexity of the Sensi-Pro analysis compared to the other GAG-analysis methods, we explored a highly simplified method. We omitted the protease, ion exchange, and desalting steps. The DBS punch was first incubated in buffer with bacterial hepara\nases, as was done for the internal disaccharide method. The sample was concentrated to dryness and dissolved and submitted to derivatization with aniline/NaCNBH_3_ in acetic acid/dimethylsulfoxide.

**Figure 7:**
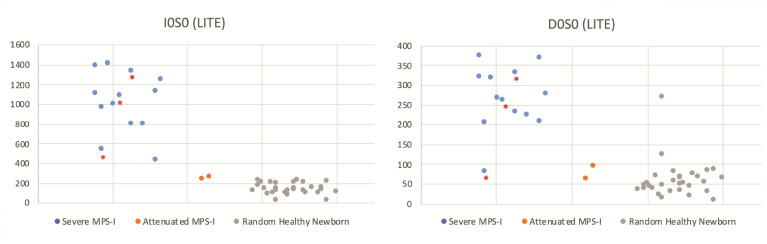

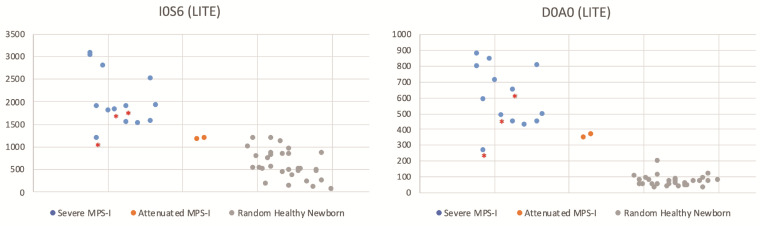
Same as Figure 6 except Sensi-Pro lite was used. See Figure 2 legend for an explanation of the singe random newborn with high biomarker levels. Asterisked dots are from newborn DBS that were stored at ambient temperature, all others were stored frozen.

Figure 7 shows the levels of Sensi-Pro Lite biomarkers in newborn DBS from 13 severe MPS-I patients, two attenuated MPS-I patients, and 30 random newborns. The levels of internal disaccharide markers (D0A0 and D0S0) measured in the same LC-MS/MS run are also shown. Values for all samples are provided in Table S3.

## 4. Discussion

In this study, we report the levels of GAG-derived biomarkers in newborn DBS from MPS-I patients. One goal of the study was to do a comparative evaluation of multiple GAG analysis methods that have been previously reported. A second goal was to expand the database of GAG levels in MPS-I DBS from newborns in particular since these samples are most relevant for the use of GAG analysis as a component of NBS, and there is very little data with newborn samples. Obtaining newborn DBS from MPS-I patients is difficult, as it requires locating families with an affected patient in states where the stored samples are still available. Over the past ~2 years and with major help from the National MPS Society, we have been able to obtain newborn MPS-I DBS including two from patients with attenuated disease. The inclusion of attenuated patients is especially important, because there is almost no prior data on GAG levels in newborn DBS from these patients. We are continuing to obtain as many samples as possible, and the current report is based on the samples we have obtained during the past ~2 years. The NBS community is justifiably unsettled about using GAG analysis as a go/no-go NBS criterion to follow first-tier measurement of IDUA enzymatic activity in DBS. There is a general sense that attenuated MPS-I patients respond very well to early treatment and should be identified as part of NBS.

The present study is limited to mass spectrometry-based GAG analysis, which is generally accepted to be far superior to the measurement of bulk levels of intact GAG polymers in urine by assays that rely on binding of cationic fluorometric dyes. Furthermore, only mass spectrometry assays can be applied to DBS and thus be incorporated into NBS. This is an important consideration given that the majority of at-risk newborns identified only by low IDUA activity in DBS are false positives, due in part to common pseudodeficiencies [8], and there is the obvious desire to reduce anxiety to families by minimizing the NBS follow-up referrals that may take weeks to collect and analyze diagnostic data.

Mass spectrometric analysis of intact GAG polymers is problematic because of the enormous number of molecular species due to chemical variation of disaccharide units in the polymer chain. Thus, all methods are based on detection of biomarkers that are short GAG fragments generated either via enzymatic or non-enzymatic depolymerization or formed endogenously in tissues. The most common method is to degrade the GAG polymer with bacterial hydrolases and lyases, and this is the basis of the internal disaccharide method described in this paper. A variation of this method is to non-enzymatically degrade the GAG polymer by partial methanolysis to generate methyl glycosides of disaccharides [16]. We did not study this method in the current study, since we had limited newborn DBS samples, and it is expected to give results similar to the internal disaccharide method using bacterial degradation enzymes. We also studied three methods that look at the non-reducing end of the GAG chain, namely Sensi-Pro and its variation Sensi-Pro Lite, and the endogenous disaccharide method, the most recent of the published methods.

With the internal disaccharide method, the heparan sulfate-derived GAG markers D0A0 and D0S0 show elevation in all MPS-I DBS tested above the reference range (Figure 2). The levels in DBS from the two attenuated MPS-I patients were lower than those in the majority of the samples from severe MPS-I patients. For the dermatan sulfate-derived marker D0a4, there was overlap in levels in the attenuated MPS-I samples with those in the reference range. Looking at the lowest level in patients and the highest level in non-patients, the fold difference (referred to as the “minimum differential” is 2.4-fold for D0A0, 3.0-fold for D0S0, and 1.5-fold for D0a4 for severe MPS-I patients. The minimum differentials for the attenuated MPS-I patients are 1.4-fold for D0A0, 0.9-fold for D0S0 (i.e., overlap with the reference range), and 0.7-fold for D0a4. Note that an outlier was seen for one of the random newborn DBS (presumably normal), which exhibited high D0A0 and D0S0 levels in both the internal disaccharide method (Figure 2) and Sensi-Pro Lite (Figure 7) is not included in these calculations of minimum differential. We suspect that the high levels of biomarkers in this random newborn may be indicative of a previously undetected enzyme deficiency, but we have no additional data about this newborn.

These results are similar to results reported previously for GAG biomarker levels in newborn DBS from MPS-I patients. Wijburg and colleagues reported data on newborn DBS from 10 MPS-I patients, including a single Scheie patient (attenuated MPS-I). The latter showed the lowest level of elevation; the minimal differential was 1.8-fold for D0A0 and ~2.7 for D0a4 (de Ruijter et al., 2012). Tomatsu and colleagues reported a minimal differential of ~1.7-fold for D0A0 and ~1.5-fold for D0S0 for a dataset of newborn DBS with three MPS-I patients and 30 non-patients; the severity of the MPS-I was not provided (Tomatsu et al., 2010). Similar trends were reported in a second study from Tomatsu and co-workers on three additional MPS-I patients [17]. Recently, the Mayo Clinic reported a large scale study of GAG biomarkers in DBS from MPS-I screen positive newborns [8]. A total of 1166 newborn DBS were analyzed. Among these, 13 showed the largest elevation of D0A0 (3- to 9-fold higher than the top of the reference range) along with elevation of D0a4 (8- to 50-fold higher than the top of the reference range). The clinical status of these patients is not known; however, the genotype was available and showed 10/13 with two pathogenic mutations, two with pathogenic/likely pathogenic, and one with pathogenic/VUS). Nine of the 1166 newborns showed a lower degree of GAG biomarker elevation, up to 2-fold and 4-fold above the top of the reference range for D0A0 and D0a4, respectively. Genotyping showed 6/9 with at least one pseudodeficiation variation, 3/9 with pseudodeficiency/VUS, and 1 with pathogenic/likely pathogenic.

The endogenous disaccharide method shows the highest disease-to-non-disease discrimination among the four methods tested in this study with a minimum differential of 12.4-fold (Figure 3). This is due to the observation that the biomarker is essentially undetectable in non-MPS-I newborn DBS. This is not the case for the internal disaccharide method where clear LC-MSMS MRM peaks were seen for all 30 non-MPS-I newborn DBS. The endogenous disaccharide method also shows clear separation between the severe and attenuated MPS-I newborn DBS (although only two attenuated samples were available for this study). An additional advantage of the endogenous disaccharide method is that no enzymatic or chemical digestion is needed and thus the pre-LC-MS/MS sample preparation time is shorted. The disadvantage is that the relatively low level of marker in MPS-I patient samples requires use of a mass spectrometry with high-end sensitivity. For this study, the endogenous markers were measured with a Sciex 6500 triple quadrupole mass spectrometer (using one or two 3 mm DBS punches). However, some investigation was done using a Waters Xevo TQ-S instrument, and this provided marker detection with similar signal-to-noise although two 3 mm DBS are needed (not shown).

Despite the fact that Sensi-Pro measures the level of GAG non-reducing end, the MPS-I/non-MPS-I discriminatory power of this method is similar to that of the internal disaccharide method using I0S0 (compare Figure 2 and Figure 6). The I0S6 marker showed very poor discrimination (Figure 6). Sensi-Pro Lite performed slightly better than Sensi-Pro for the I0S0 marker, and performed much better than Sensi-Pro for the I0S6 marker (compare Figure 6 and Figure 7). Again, a key advantage of the endogenous disaccharide method is the virtual absence of biomarker MS/MS signal for non-MPS-I DBS.

Only for Sensi-Pro and Sensi-Pro Lite were we able to use a chemically-identical, isotopically-distinguished internal standard (I0S0 and I0S6 derivatized with deuterated aniline). The other two methods make use of the structural analogs chondrosine and chondroitin disaccharide di-4S as the internal standards. Thus, for these two methods, the moles of biomarker reported are apparent values that different from the true values by a constant, the value of which is unknown (due to differential mass spectrometer response factors for analyte and internal standard). Many laboratories calibrate their GAG analysis methods by using commercially available GAG polymers or disaccharide standards made by enzymatic digestion of GAG polymers. However, these materials are not available as certified reagents where the absolute abundance of material is accurately determined. These materials are likely not pure by weight, and thus reliable measurement of moles by gravimetric methods is not possible (see Gelb, 2018 for a detailed discussion of this issue) [18]. In the case of the methanolysis method, chemically identical, isotopically labeled internal standards are made available by degradation of the GAG polymer using deuterated methanol [16] but again the absolute amount of GAG polymer is not known a priori, nor is the absolute moles of internal standard known after methanolysis. These are very important issues that are usually not considered in GAG analysis in biochemical genetics laboratories, and they make it virtually impossible to compare reported absolute GAG abundance levels between different reference laboratories. This implies that each reference laboratory will have to determine their own disease-specific reference ranges, which is not very practical given the difficulty in obtaining newborn DBS from MPS-I patients, especially from those with attenuated disease. Work is in progress in the author’s laboratory to make available a certified internal standard for use in the endogenous disaccharide method.

All of the newborn DBS were stored frozen in the state NBS lab except for three stored at ambient temperature (indicated by asterisks in Figure 2 and Figure 4, Figure 5, Figure 6, Figure 7). The data shows a slight tendency of MPS-I marker instability for ambient-stored DBS, but there is insufficient data to make a firm conclusion. Loss of the MPS-II marker with ambient temperature storage may be a bigger factor (Figure 5).

The present study is focused on MPS-I, which is the highest priority given that NBS for this disease has started in several NBS laboratories. The study is being continued using DBS from newborns with other forms of MPS disease and results will be presented when available.

## Figures and Tables

**Table 1:** Comparison of four glycosaminoglycans (GAG)-analysis methods.

Method	Biomarkers	Sample	Sample Preparation	Quantification Method
Internal disaccharide	D0A0, D0S0, D0a4	1 × 3 mm DBS punch	Enzymatic digestion with heparinases and chondroitinase B	hydrophilic-LC-MS/MS
Endogenous disaccharide	UA-HNAc(1S)	1 or 2 × 3 mm DBS punch	Derivatization with PMP	hydrophobic-LC-MS/MS
Sensi-pro	I0S0, I0S6	1 × 3 mm DBS punch	Protein digestion with pronase, GAG digestion with heparinases, derivatiztion with aniline/NaCNBH_3_	hydrophobic-LC-MS/MS
Sensi-pro Lite	I0S0, I0S6	1 × 3 mm DBS punch	GAG digestion, with heparinases, derivatiztion with aniline/NaCNBH_3_	hydrophobic-LC-MS/MS

## Supplementary Materials

The following are available online at https://www.mdpi.com/2409-515X/6/3/69/s1.
