# Supplementary material for: Evaluation of Multiple Methods for Quantification of Glycosaminoglycan Biomarkers in Newborn Dried Blood Spots from Patients with Severe and Attenuated Mucopolysaccharidosis-I
Source: Int J Neonatal Screen. 2020 Aug 26;6(3):69. doi: 10.3390/ijns6030069 (PMC7570209; doi:10.3390/ijns6030069)

Supplementary Material

Supplementary Table S1. MPS-I patient information provided by parents on 13 severe MPS-I patients and 2 attenuated MPS-I patients (last two table entries)

sex	Age at diagnosis	Age at first Symptoms	First symptoms	Current age	Current symptoms	genotype	DBS storage
F	1.1 yr	0.7 yr	Ear infect.<br>Kyphosis	1.7 yr	Kyphosis<br>Gross motor delay<br>Hearing loss<br>Corneal clouding	p.Tyr344*<br>c.1205G>A/<br>pTrp402*	1.7 yr<br>ambient
M	0.6 yr	0.4 yr	Bump on back<br>Chronic breathing issues	1.3 yr	Hearing loss	?	1.3 yr<br>frozen
M	0.7 yr	0.01 yr	Enlarged cornea<br>Ear infect.<br>Hernia (groin)	16 yr	Corneal clouding<br>Joint stiffness<br>Heart valve regurg.	T388R/R621X	16 yr<br>frozen
M	1.3 yr	1.3 yr	Enarged head and tongue<br>Umbilical hernia	3 yr	Heart issues<br>High blood pres.<br>Umbilical hernia<br>Corneal clouding	?	3 yr<br>ambient
M	1.2 yr	1 yr	Enlarged head<br>Kyphosis<br>Coarse facial features	3 yr	Kyphosis<br>Corneal clouding<br>Develop/Speech delay	?	3 yr<br>ambient
F	0.1 yr	?	Cardiac issues	0.8 yr	Small curve in spine	?	0.7 yr<br>frozen
F	0.8 yr	0.3 yr	Enlarged head<br>Flared out, bell-shaped ribs	3.3 yr	Hydrocephalus<br>Heart valve regurg.<br>Low muscle tone<br>Nasal congest.<br>Knocked knees	?	3.4 yr<br>frozen
M	1.4 yr	1.3 yr	Bump on spine<br>Kyphosis<br>Corneal clouding<br>Thick/harrow fingers<br>Flat bridge nose	26.6 yr	Joint stiffness<br>Walking diff.<br>Learning disabil.	?	26.5 yr<br>frozen
M	1.3 yr	0 yr	Hearing loss<br>Behind in milestones<br>Leaky mitral valve<br>Enlarged head<br>Sleep Apnea<br>Enlarged tonsils	2.8 yr	Speech delay<br>Kyphosis<br>Enlarged tonsils<br>Corneal clouding<br>Hearing loss<br>Sleep apnea<br>Phys. delays	c.1205 G>A/ c.1598 C>G	2.8 yr<br>frozen

M	0.9 yr	0.7 yr	Weakness<br>Not meeting milestones	decease d<br>1999	?	?	20 yr<br>frozen
F	6 yr	4 yr	Range of motion issues<br>Strabismus<br>Hernia<br>Pectus caveus	21.3 yr	Hernia<br>Heart valve stenosis<br>Corneal clouding	?	21.2 yr<br>frozen
F	0.7 yr	0 yr	Lung congestion	decease d 2013	Cardiac issues	W402X/?	8 yr<br>frozen
F	3 yr	1.3 yr	Umbilical hernia<br>Freq. colds<br>Ear infect.<br>Joint stiffness	?	?	?	?<br>frozen
M	15.5 yr	0 yr	Premat.<br>Lung issues<br>Hernia<br>Ear infect.<br>Joint contractures	32.1 yr	Speech delay<br>Kyphosis<br>Enlarged tonsils<br>Corneal clouding<br>Constipation<br>Hearing loss<br>Sleep apnea<br>Phys. delays	W402X/int 11-7C>T	32 yr<br>frozen
F	9.5 yr	0.8 yr	Perri-robin syndr.<br>Ear infect.<br>Hernia<br>Joint contractures	28.1 yr	Hernia<br>Spinal issues<br>Cardiac issues	W402X/int 11-7C>T	28 yr<br>frozen

Supplementary Table S2. GAG-derived saccharides and their names.

**Compound**	**Structure**	**Name 1** (Lawrence et al., 2008)	**Name 2** (Stapleton et al., 2020)	**Assay**
Dermatan sulfate/Chondroitin sulfate B internal disaccharide	ΔUA-GalNAc(4S)	D0a4	Chondro ΔDi-4s	Internal disaccharide
Heparan sulfate internal disaccharide	ΔUA-GalNAc	D0A0	HS-0S	Internal disaccharide
Heparan sulfate internal disaccharide	ΔUA-GalN(S)	D0S0	HS-NS	Internal disaccharide
Heparan sulfate non-reducing end disaccharide	IdoA-GlcNS	I0S0	n/a	Sensi-Pro Sensi-Pro Lite
Heparan sulfate non-reducing end disaccharide	IdoA-GlcNS(6S)	I0S6	n/a	Sensi-Pro Sensi-Pro Lite
Heparan sulfate non-reducing end disaccharide	UA-HNAc(1S)	n/a	n/a	Endogenous disaccharide

Supplementary Table S3. Endogenous disaccharide marker (UA-HNAc(1S) in MPS urine and fibroblasts. Shown are apparent µmole per mole of creatinine (urine) and apparent fmole per mg protein (fibroblasts). GM numbers for fibroblasts are the sample numbers from the Coriell Institute Cell Repository*.

Urine from Dr. Fuller µmol/mol creatinine	MPS-I Marker *UA-HNAc(1S)*	Fibroblasts from Coriell fmol/mg protein	MPS-I Marker *UA-HNAc(1S)*
Healthy	0	Healthy	(not tested)
MPS-I	294	MPS-I	2456
MPS-II	1	MPS-II	36
MPS-IIIA	4	MPS-IIIA	65
MPS-IIIB	1	MPS-IIIB	85
MPS-IIIC	1	MPS-IIIC	(not tested)
MPS-IIID	1	MPS-IIID	(not tested)
MPS-IVA	1	MPS-IVA	70
MPS-IVB	0	MPS-IVB	(not tested)
MPS-VI	1	MPS-VI	75
MPS-VII	3	MPS-VII	45

*Coriell Fibroblasts References: MPS-I (GM00798), MPS-II (GM01929), MPS-IIIA (GM00312), MPS-IIIB (GM00156), MPS-IVA (GM00593), MPS-VI (GM00538), MPS-VII (GM02784)

Supplementary Table S4. Individual values of biomarker levels for Sensi-Pro and Sensi-Pro lite for all DBS samples.

Sample Name	Sensipro Lite Results				Sensipro Full Results			
	I0S0	I0S6	D0S0	D0A0	I0S0	I0S6	D0S0	D0A0
	pg/punch	pg/punch	apparent pg/punch	apparent pg/punch	pg/punch	pg/punch	apparent pg/punch	apparent pg/punch
MPS-I (#106) - Severe	1383	3027	321	878	759	517	2214	2122
MPS-I (#112) - Severe	1113	3054	375	801	802	601	2261	1932
MPS-I (#115) - Severe	974	1898	206	588	718	915	1896	2126
MPS-I (#117) - Severe	1413	2793	319	843	1214	689	2263	2084
MPS-I (#118) - Severe	1128	2505	370	806	422	494	2040	1931
MPS-I (#122) - Severe	1004	1792	268	714	527	1133	2055	2213
MPS-I (#400) - Severe	794	1541	234	447	683	462	2096	2038
MPS-I (#500) - Severe	1336	1887	332	648	848	879	2177	2228
MPS-I (#501) - Severe	1082	1813	263	489	862	685	2204	2140
MPS-I (#604) - Severe	1249	1909	277	495	687	844	2065	1910
MPS-I (#605) - Severe	427	1559	207	450	365	475	2229	1995
MPS-I (#607) - Severe	802	1523	224	430	622	573	1527	1634
MPS-I (#700) - Severe	535	1191	82	272	492	340	2142	2055
MPS-I (#120) - Attenuated	234	1169	65	347	273	108	2035	1825
MPS-I (#119) - Attenuated	264	1185	96	366	258	149	1958	1709
Random Newborn	103	497	76	49	174	371	1628	1578
Random Newborn	133	478	85	95	163	455	1642	1498
Random Newborn	119	500	46	47	181	389	1559	1407
Random Newborn	152	452	30	34	162	440	1688	1529
Random Newborn	227	523	39	52	151	692	1633	1470
Random Newborn	104	112	54	76	111	333	1576	1389
Random Newborn	200	563	48	53	165	697	1847	1625
Random Newborn	202	469	20	64	155	654	1780	1659
Random Newborn	143	532	46	92	171	520	1548	1481
Random Newborn	111	52	67	80	132	440	29	26
Random Newborn	92	494	39	81	146	295	1533	1532
Random Newborn	206	169	72	54	109	712	1635	1641
Random Newborn	155	222	69	71	135	554	618	1149
Random Newborn	142	474	49	72	166	556	1403	1559
Random Newborn	206	133	35	57	136	640	1566	1398
Random Newborn	226	372	54	43	148	676	1512	1482
Random Newborn	19	245	11	71	138	23	1910	2036
Random Newborn	83	424	59	54	156	337	1524	1416
Random Newborn	94	1120	33	43	292	312	1724	1461
Random Newborn	138	1186	269	203	234	394	1947	1720
Random Newborn	115	988	38	105	260	406	1465	1406
Random Newborn	137	961	66	86	214	427	1899	1666
Random Newborn	175	1185	49	83	262	584	2036	1838
Random Newborn	118	860	125	114	209	441	1914	1707
Random Newborn	133	834	69	78	210	381	1790	1564
Random Newborn	19	802	16	55	188	18	1970	1826
Random Newborn	204	797	53	54	198	675	1850	1627
Random Newborn	121	828	83	74	219	383	1944	1706
Random Newborn	217	858	88	123	211	728	1877	1793
Random Newborn	96	734	23	35	224	343	858	1114

Supplementary Figure S1. Internal disaccharide method with digestion of GAGs with heparinases in Tris buffer (left) or Trist buffer + Ca^{2+} + dithiothreitol (DTT) (additional details in Methods). Because of limited DBS samples, DBS from attenuated MPS-I patients were not included in the study with Ca^{2+} + DTT. Plotted is apparent pmoles biomarker per 3 mm DBS punch (apparent, because it is based on non-chemically identical chondrosine as an internal standard).

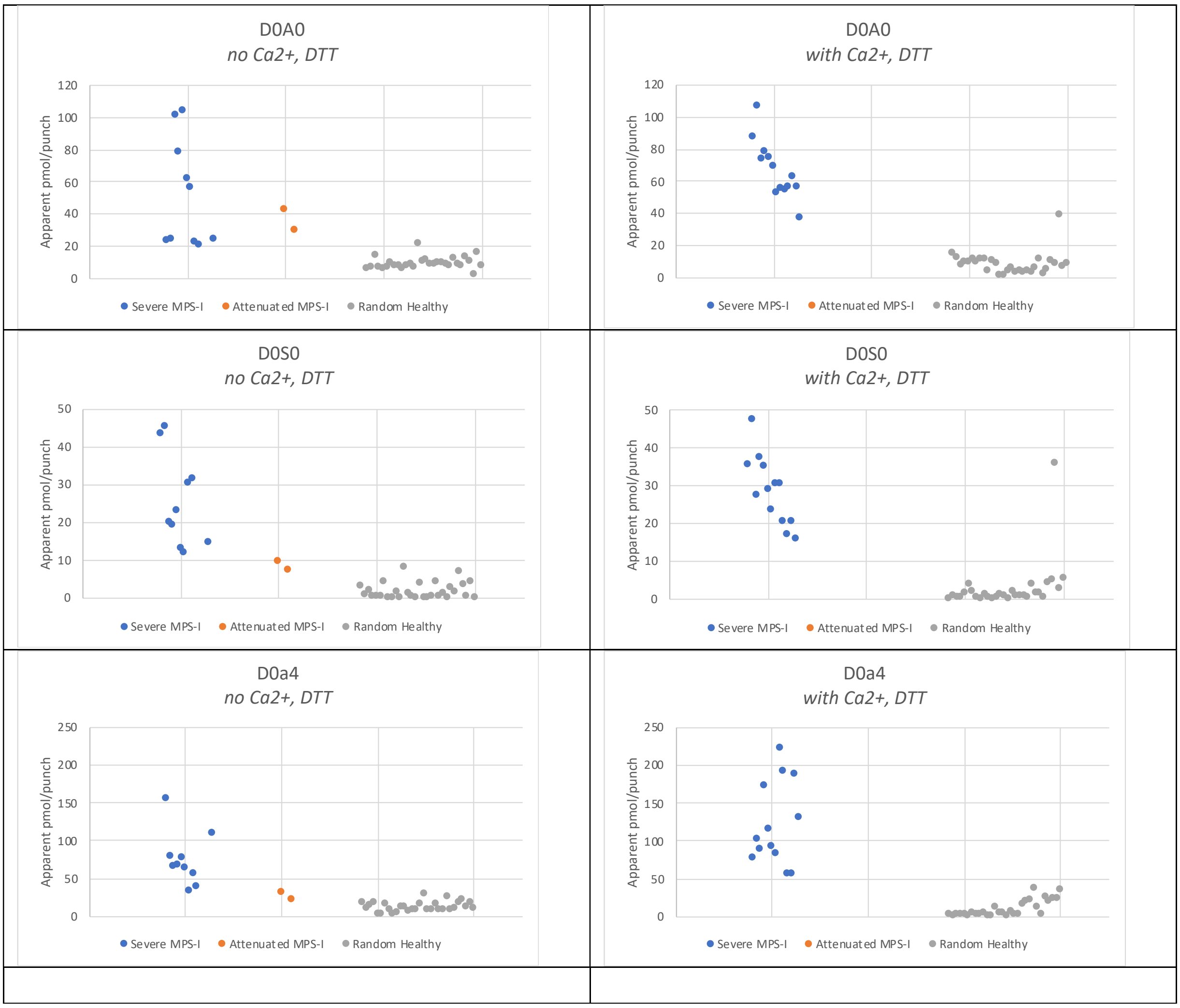

Supplementary Figure S2.

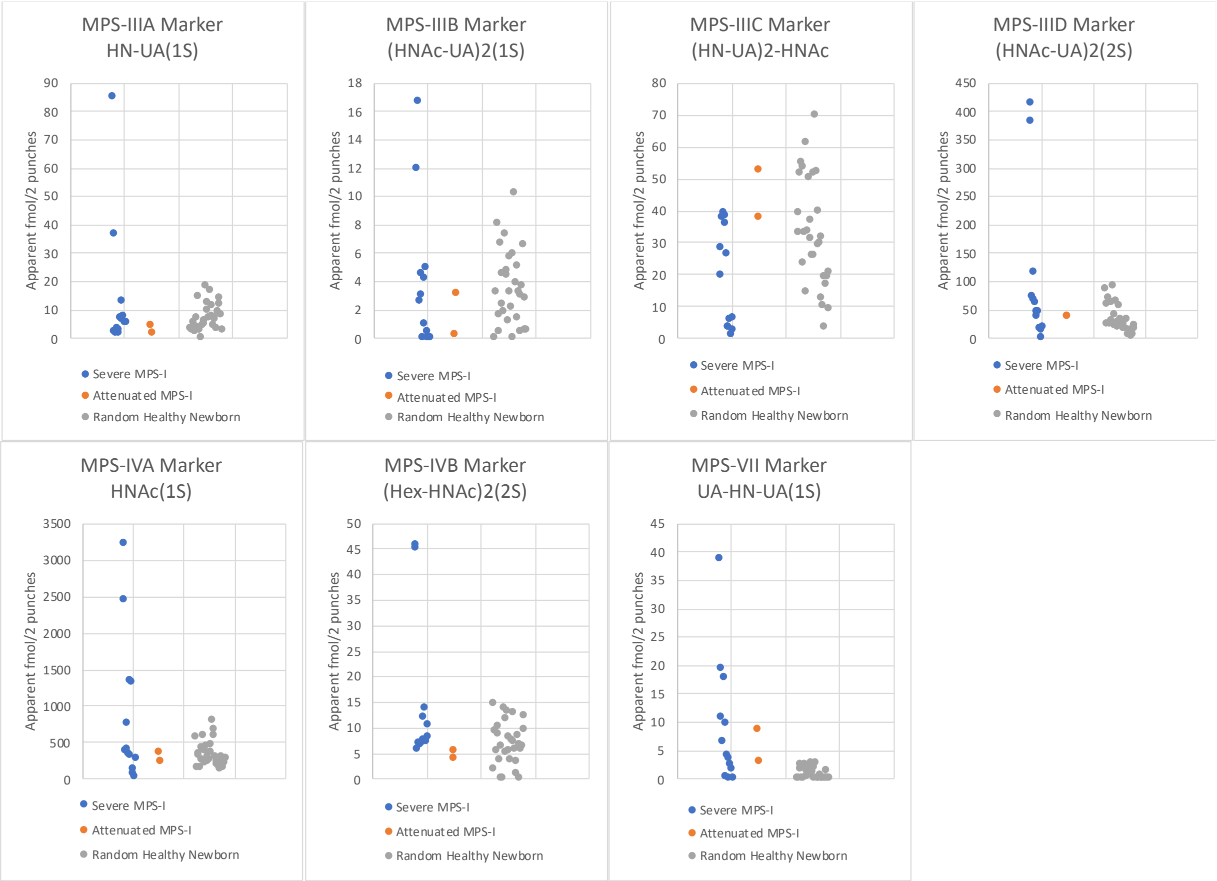

Supplementary Figure S3. I0S6 detected by Sensi-pro in newborn DBS from three severe MPS-I patients.

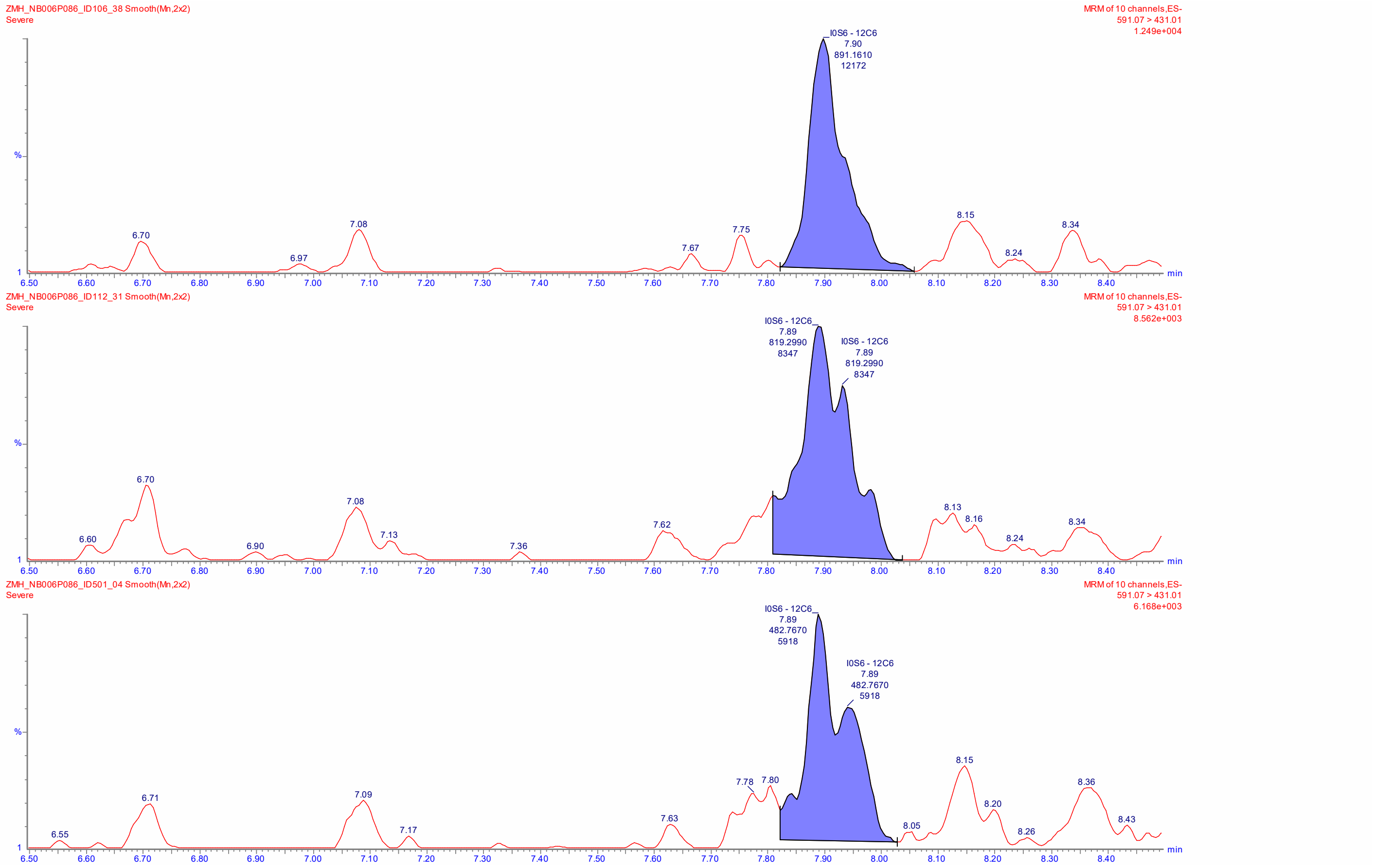

Supplementary Figure S4. I0S0 and I0S6 Sensi-pro markers detected by LC-MS/MS using the I0S0 MRM channel in newborn DBS from three severe MPS-I patients.

I0S6 Marker (in I0S0 channel) *These peaks were used in results. Integration parameters set in Waters TargetLynx to measure from the baseline, located nearest peak to predicted retention time (+/- 0.1 min), auto-selection of peak width with ApexTrack, peak smoothing enabled, peak-to-peak baseline noise = 10, peak width at 5% height = 30,*

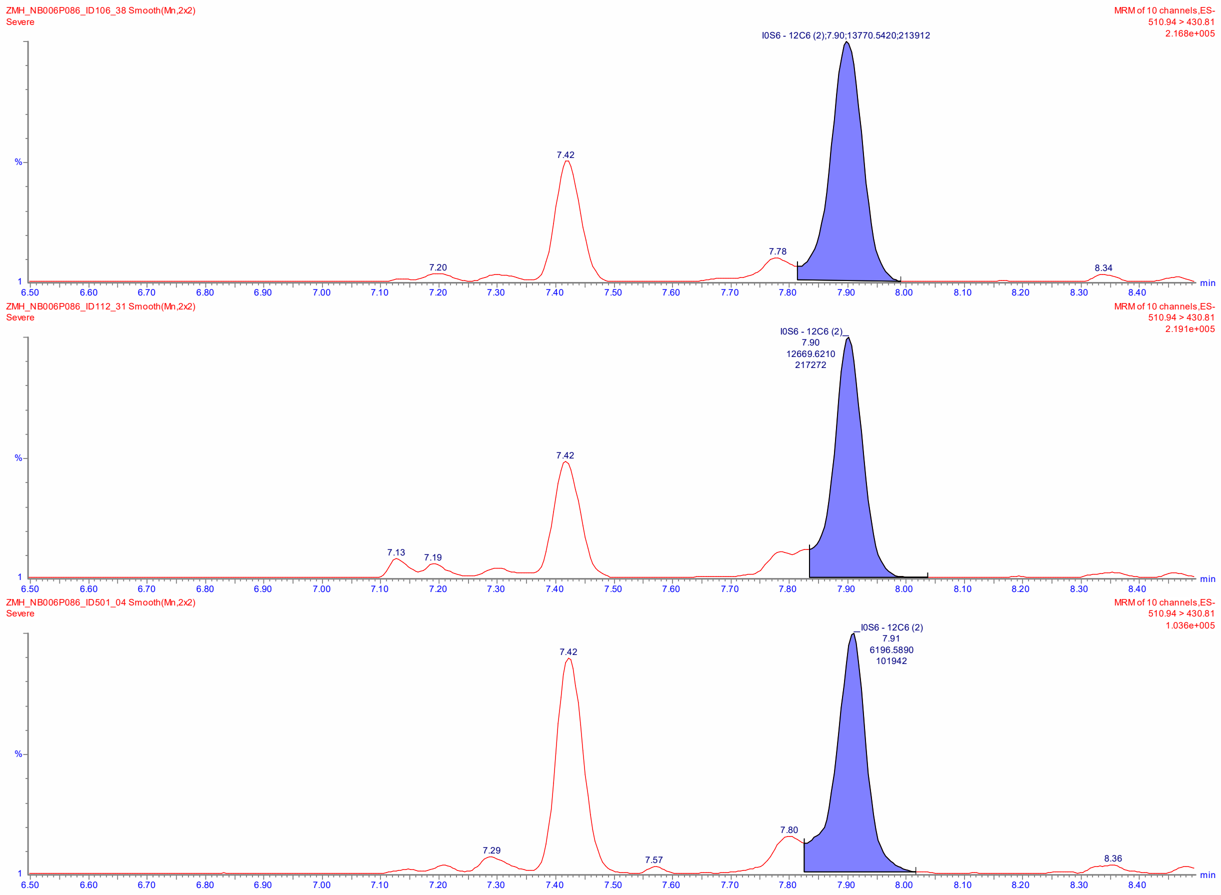

STANDARD OPERATING PROCEDURES

Version: 07-13-2020
SOP FOR DETERMINATION OF GAG LEVELS IN MPS-I PATIENT SAMPLES VIA ENZYME DIGESTION AND LC-MS/MS (INTERNAL DISACCHARIDE METHOD)

MATERIALS

Samples:
- Donated anonymous healthy newborn dried blood spots (DBS) (from Dr. Francyne Kubaski, Universidade Federal do Rio Grande, Brazil and Dr. Zoltan Lukacs, University of Hamburg, Germany)
- Donated anonymous MPS-I newborn dried blood spots (DBS) (from the National MPS Society, United States)

Standards and Reagents:
- Internal Standard (IS), "Chondrosine" 2-Amino-2-deoxy-3-O-(b-D-glucopyranuronosyl)-D-galactopyranose (CAS 499-14-9) (Carbosynth, Ref: OA10113)
- Chondroitin/Dermatan Sulfate Disaccharide "D0a4" (CAS 136144-56-4) (Iduron, Ref: CD002)
- Heparan Disaccharides Mixture "D0A0" and "D0S0," I mL of 75 nM (Iduron, Ref: HD Mix)
- Chondroitinase B from *Flavobacterium heparinum*, 50 UN (Sigma, Ref: C8058-50UN, 1UN* = 0.1 µmole/hr)
- Heparinase I from *Flavobacterium heparinum*, 0.5 IU, lyophilized (Ibex, Ref: 60-400, 1IU* = 1.0 µmole/min)
- Heparinase II from *Flavobacterium heparinum*, 0.5 IU, lyophilized (Ibex, Ref: 60-400, 1IU* = 1.0 µmole/min)
- Heparinase III from *Flavobacterium heparinum*, 0.5 IU, lyophilized (Ibex, Ref: 60-400, 1IU* = 1.0 µmole/min)
- Bovine Serium Albumin (BSA) lyophilized powder (Sigma, Ref: A0281-10G)
- TRIS Ultra-Pure Grade (Amresco, Ref: 201-064-4)
- Dithiothreitol (DTT) (CAS 3483-12-3) (Sigma Aldrich, Ref: D0632)
- Calcium chloride (CAS 7440-70-2) (Sigma Aldrich, Ref: C-3306)
- Methanol, HPLC Grade (CAS 67-56-1) (Fisher, Ref: A452-1)
- Ammonia Solution 28-30% (CAS 1336-21-6) (MilliporeSigma Ref: 105423)
- Methanol, Optima Grade (CAS 67-56-1) (Fisher, Ref: A456-4)
- Water, Optima Grade (CAS 7732-18-5) (Fisher, Ref: W6500)

** 1.0 International Unit (IU) = 600 "Sigma" Units (UN) (check definition of units on company spec sheets to be sure)*

Benchtop Supplies:
- Deep-well 96-well plate, polypropylene, round bottom, 1mL well (Costar, Ref: 3959)
- Sealing film for 96-well plate (Axygen, Ref: PCRSP)
- MilliQ Water Filtration System
- N2 stream evaporator with 96-well plate adapter (made in-house)
- Allegra X-12R Centrifuge (Beckman Coulter)

Mass Spectrometry Supplies/Equipment:
- Shallow-well 96-well plate, polypropylene, v-bottom, 350 µL well (Greiner Bio-One, Ref: 651201)
- Hypercarb porous graphitic carbon column, 50 x 2.1 mm, 5 µm (Thermo, Ref: 35005-052130)
- Hypercarb guard columns, 10 x 2.1 mm, 5 µm (Thermo, Ref: 35005-012101)
- Universal uniguard pre-column holder (Thermo, Ref: 852-00)
- Waters Xevo TQ-S Mass Spectrometer with Water's I-Class Acquity UPLC with flow-through needle injector

METHODOLOGY

Prepare the following solutions:

- 50 mM Tris Buffer, 13.2 mM Calcium Chloride, pH 7.0. Dissolve 181.7 mg Tris base and 36.6 mg calcium chloride in 10 mL MilliQ water. Adjust the pH with HCl and bring volume to 30 mL with MilliQ water. Store at 4°C.
- 100 mM DTT stock in water: Dissolve 15 mg DTT per 1.00 mL of MilliQ water. Prepare fresh before each assay.
- 1% BSA in water: Dissolve 0.5 g BSA in 50 mL MilliQ water. Store at 4°C.
- 0.1 mM Chondrosine (IS) stock in water: Use qNMR to determine the exact amount of chondrosine you are starting with. For each 1 mg of chondrosine (355.3 g/mol), add 281.5 µL MilliQ water to make 10 mM solution. With a Hamilton Syringe, dilute 5 µL of the 10 mM stock to 500 µL using MilliQ water to make 0.1 mM stock. Aliquot the 0.1 mM stock into vials with Teflon-septum screw caps and store all stocks at -20°C. Seal the vials with Parafilm to prevent evaporation.
- 1 mg/mL (1.99 mM) Doa4 in water: Make 1 mg/mL stock by adding 1 mL MilliQ water to 1 mg of Doa4 powder. This is not quantified by qNMR.
- 75 nM DoA0 and DoS0 stock: Use HD Mix stock as received from the vendor. This is not quantified by qNMR.
- MS-STD Solution: Prepare enough MS-STD solution for 200 response factor checks, or 2 mL. Prepare the standard by combining 20.0 µL of 10 mM Chondrosine (IS), 20.1 µL of 1 mg/mL (1.99 mM) Doa4, 533.3 µL of HD Mix (75 nM), and 1,191.1 µL MilliQ water. Per each set of assays, 10 µL of this stock will be added to a blank well to ensure that the response of each marker is proportional to the response of the IS as compare to the previous assays (see below).
- Heparinase I, II, and III stocks*: Dissolve Heparinase I, II, and III (0.5 IU) in 0.2 mL of 1% BSA in water. This is a final concentration of 2.5 IU/mL. Aliquot into 10 µL fractions and store at -20°C to avoid multiple freeze-thaw cycles. More dilute solutions may be made if less than 20 samples will be run at a time.
- Chondroitinase B stock*: Make 66.7 mIU/mL stock by diluting 83 mIU (50 UN) to 1244.4 µL using 1% BSA in MilliQ water. Aliquot into 10 µL fractions and store at -20°C to avoid multiple freeze-thaw cycles.

  ** Enzymes seem to be stable with up to 3 freeze-thaw cycles, so make aliquots such that you can keep the number of cycles to 3, discarding after 3 cycles.*

  NOTE: We will assume that all MSMS response factors are unity relative to chondrosine. That is, if you inject the same mole amount of disaccharide and chondrosine, you would get an MSMS intensity of the disaccharide divided by the MSMS intensity of chondrosine of unity (equals 1). This is not generally true, but we assume it in this analysis. This means the moles of disaccharide reported are apparent values relative to chondrosine and not true values. The fold change in disaccharide level will be true since the response factor cancels out of such calculations. This assumes that the disaccharide to chondrosine response factor does not change with time. We don't have stocks of disaccharide standards of known mole amount, only the stock of chondrosine is known in actual moles since it was standardized by qNMR. However, if the response factor measured for each disaccharide using the MS-STD solution (see stock preparation above) does not change in time, it means that our apparent calculations of moles of disaccharide are constant in time. To achieve this, we will include a well of MS-STD in each plate of GAG samples analyzed on any given date. We will keep track of the response factors of the disaccharides in MS-STD relative to chondrosine also in this sample.

Punch one 3 mm DBS disk per patient and put the punches into a well of a 1 mL deep well 96-well plate (Costar Ref: 3959). To each well containing DBS punches, add a cocktail mixture of enzymes, buffer, and internal standard:

90 μL of 50 mM Tris HCl with 11 mM $CaCl_2$, pH 7.0 (Amresco Ref: 0497)
3 μL of 100 mM DTT solution in water
10 μL of 0.1 mM Chondrosine (IS), 1 nmole per well
10 μL of 66.7 mIU/mL chondroitinase B, 0.667 mIU per well
10 μL of 100 mIU/mL heparinase I, II, III, mixed, 1.0 mIU of each heparinase per well

Seal the plate with sealing film and incubate at 37°C while shaking at 250 rpm. After overnight incubation (16h ± 1h), quench each sample with 400 µL of cold methanol, pipetting up and down 20 times to mix, and incubate over ice for 10 minutes to precipitate proteins. Centrifuge the plate for 15 min at 3,000 *g*. Transfer 300 µL of supernatant to a new v-bottom 96-well microplate (Griener Bio-One Ref: 651201) and concentrate to dryness with N2 stream. Reconstitute samples in 100 µL water and mix up and down 20x with a pipette. Measure Doa4, DoA0, and DoS0 levels via UPLC-MS/MS.

To check the relative response of markers to IS, prepare a blank well with 100 µL of Tris buffer (no chondrosine or enzymes) and spike with 10 µL of MS-STD. Inject this standard into the MS prior to analyzing samples to ensure that there has been no drift in the response between the chondrosine (IS) and the markers (D0a4, D0A0, D0S0) since the last assay. Use the same solution of MS-STD for each assay so that the relative responses may be compared between assays. If a new solution of MS-STD must be made (when the old solution begins to run out), inject the old solution alongside the new solution so that the new solution's relative ratios can be verified before being used as a standard for future assays. Note, everything relates back to the chondrosine MSMS signal which is based on the qNMR-standardized stock solution of chondrosine IS. See the note above about response factors.

The new levels of GAG markers will be measured relative to the response of 1 nmole chondrosine per 1 DBS punch. The response ratio of each marker from an MPS patient's DBS will be recorded and compared to the DBS of healthy patients in order to determine if the level of marker meets a cut-off level. These cut-off values will need to be re-determined if the method is transferred to another MS instrument.

LC-MSMS Analysis:

Disaccharides were separated using a 5 µm 50 x 2.1mm Hypercarb porous graphitic carbon column (Thermo, P/N 35005-052130) fitted with a Hypercarb 5 µm 10 x 2.1mm guard column (Thermo, P/N 35005-012101) in a universal uniguard holder (Thermo, P/N 852-00) connected to a Waters I-Class Aquity UPLC with a flow-through needle. The column was held at 60°C. The disaccharides were eluted in a 5-minute UPLC gradient program with 148 mM ammonia in water (MPA) and 100% acetonitrile (MPB). See the program in the table below. A strong needle wash of 100% acetonitrile, a purge wash of MPA, and a seal wash of 90/10 water/acetonitrile were used.

UPLC Solvent Gradient Program:

Time (min)	Flow (mL/min)	%MPA	%MPB
0.00	0.7	100	0
1.00	0.7	50	50
2.00	0.7	50	50
2.20	0.7	0	100
2.60	0.7	0	100
2.61	0.7	100	0
5.00	0.7	100	0

Disaccharides were detected with a Waters Xevo TQ-S mass spectrometer with an ESI source in negative mode. See mass spec parameters and MRMs below. Waters MassLynx Software and TargetLynx was used to analyze the data.

Xevo TQ-S Parameters

Parameter	Setting
Polarity	ESI-
Capillary (kV)	2.0
Source Temperature (°C)	150
Desolvation Temperature (°C)	550
Cone Gas Flow (L/hr)	150
Desolvation Gas Flow (L/hr)	1200
Collision Gas Flow (mL/min)	ON
Collision Gas	Argon

Marker Names, Retention Times, MRMs, and Tuning Information for Xevo TQ-S

Marker	GAG	Retention Time (RT)	MRM	Cone (V)	Collision
Chondrosine	Internal Standard	0.69 min	355.09 > 194.07	30	14
DoAo	Heparan Sulfate	0.76 min	378.18 > 175.13	30	14
DoSo	Heparan Sulfate	0.82 min	416.11 > 138.08	70	24
Doa4	Dermatan Sulfate	0.67 min	459.2 > 300.94	66	20

EXAMPLE CHROMATOGRAMS (next page):

MPS-I (ID: 106) Newborn DBS

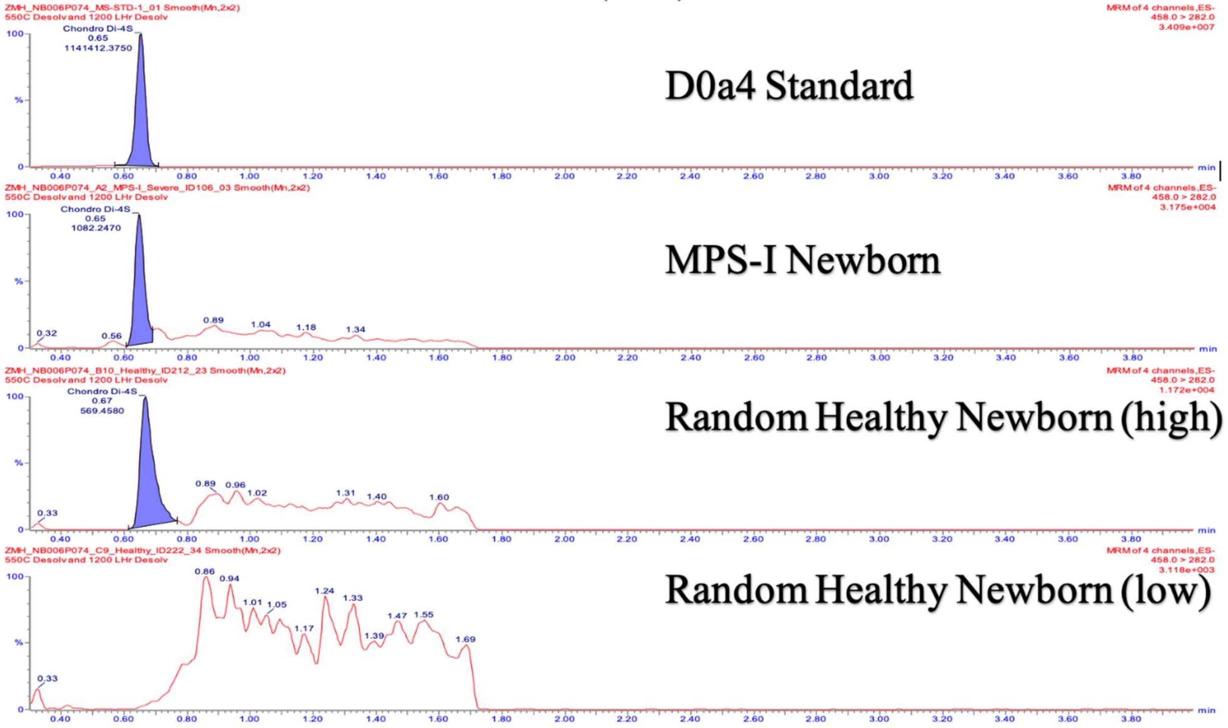

Marker	MPS-I Newborn	Random Newborn 1	Random Newborn 2
D0a4	77 pmol/punch	36 pmol/punch	0 pmol/punch

MPS-I (ID: 106) Newborn DBS

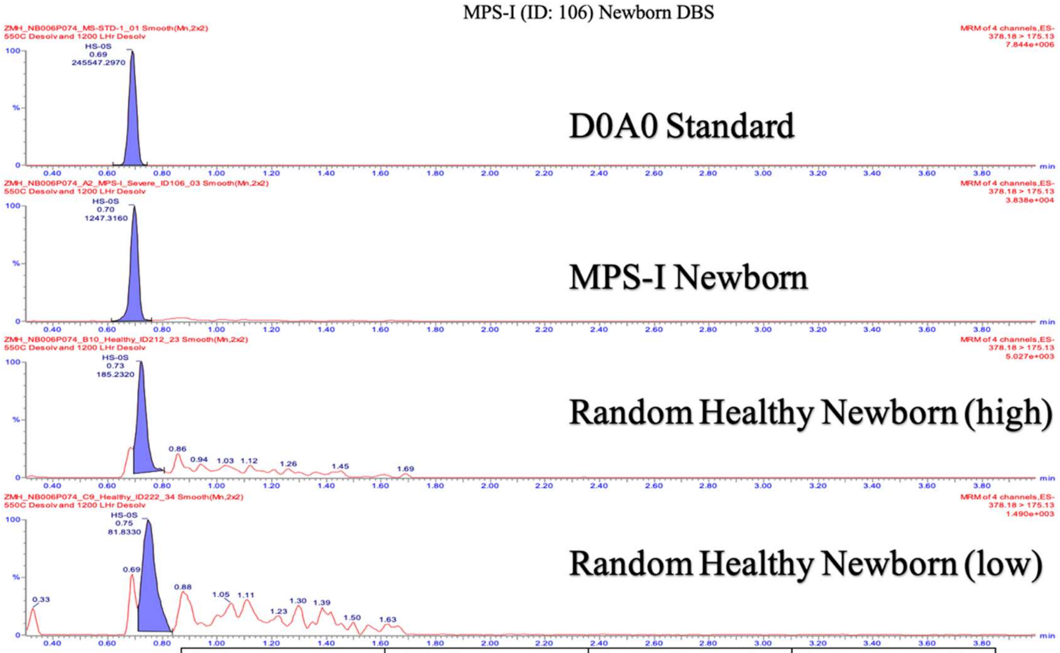

Marker	MPS-I Newborn	Random Newborn 1	Random Newborn 2
D0A0	88 pmol/punch	12 pmol/punch	9 pmol/punch

MPS-I (ID: 106) Newborn DBS

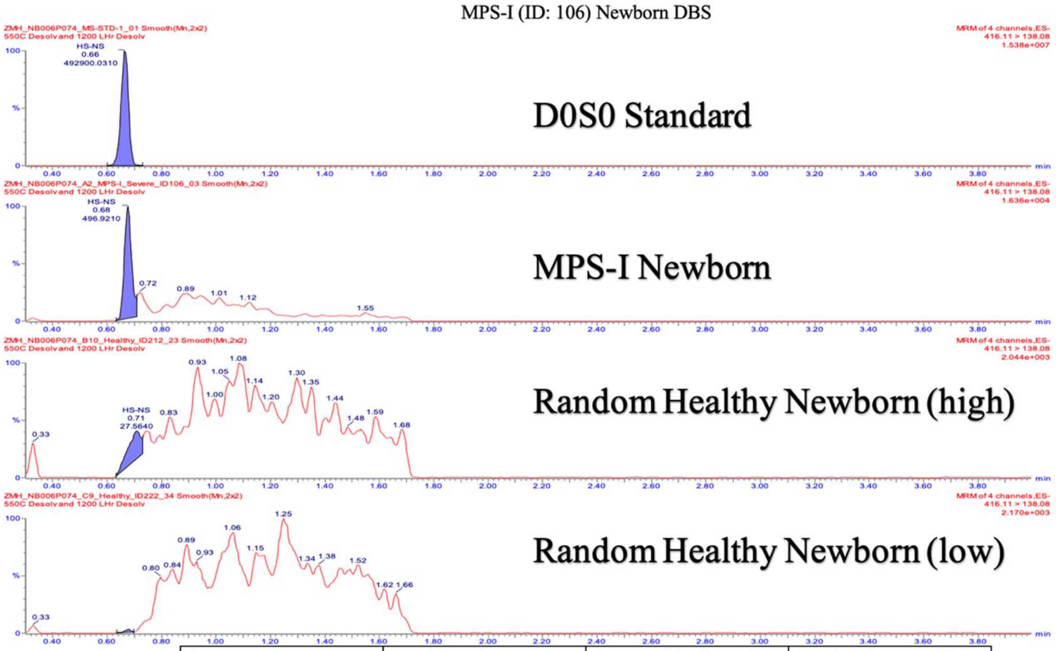

Marker	MPS-I Newborn	Random Newborn 1	Random Newborn 2
D0S0	35 pmol/punch	2 pmol/punch	0 pmol/punch

SOP FOR DETERMINATION OF GAG LEVELS IN MPS-I PATIENT SAMPLES VIA PMP-DERIVATIZATION OF OLIGOSACCHARIDES AND LC-MS/MS (ENDOGENOUS DISACCHARID METHOD)

MATERIALS

Samples:
- Coriell Institute MPS Patient Fibroblasts: MPS-I (GM00798), MPS-II (GM01929), MPS-IIIA (GM00312), MPS-IVA (GM00593), MPS-VI (GM00538), MPS-VII (GM02784)
- Donated anonymous healthy patient urine sample
- Donated anonymous MPS patient urine samples dried on filter paper (from Dr. Maria Fuller, University of Adelaide, Australia): MPS-I, MPS-II, MPS-IIIA, MPS-IIIB, MPS-IIIC, MPS-IIID, MPS-IVA, MPS-IVB, MPS-VI, MPS-VII
- Donated anonymous healthy newborn dried blood spots (DBS) (from Dr. Francyne Kubaski, Universidade Federal do Rio Grande, Brazil and Dr. Zoltan Lukacs, University of Hamburg, Germany)
- Donated anonymous MPS-I newborn dried blood spots (DBS) (from the National MPS Society, United States)

Standards and Reagents:
- Internal Standard (IS), Chondroitin Disaccharide di-4S (CAS 136144-56-4) (Carbosynth, Ref: OC28898)
- PMP, 3-Methyl-1-phenyl-2-pyrazoline-5-one (CAS 89-25-8) (Aldrich, Ref: M70800)
- Ammonia Solution 28-30% (CAS 1336-21-6) (MilliporeSigma Ref: 105423)
- Methanol, HPLC Grade (CAS 67-56-1) (Fisher, Ref: A452-1)
- Formic Acid, Optima Grade (CAS 64-18-6) (Fisher, Ref:A117-50)
- Chloroform, HPLC Grade (CAS 67-66-3) (Fisher, Ref: C606-1)
- Acetonitrile, Optima Grade (CAS 75-05-8) (Fisher, Ref: A955-500)
- Water, Optima Grade (CAS 7732-18-5) (Fisher, Ref: W6500)
- 1x DPBS (ThermoFisher, Ref: 14190144)

Benchtop Supplies:
- Pierce™ BCA Protein Assay Kit (ThermoFisher, Ref: 23225)
- pH Test strips, 4.5-10.0 pH (MilliporeSigma, Ref: P4536)
- Microtubes with snap caps, 1.5 mL polypropylene (VWR, Ref: 89000-028)
- Thin-walled PCR tubes, 0.5 mL (Sigma Aldrich, Ref: CLS6530)
- Peltier Thermal Cycler, PTC-200 (MJ Research, Ref: 8252-30-0001)
- MilliQ Water Filtration System
- SpeedVac, Vacuum Concentrator
- Benchtop Centrifuge

Mass Spectrometry Supplies/Equipment:
- Pulled-point glass HPLC vial inserts, 250 µL (Agilent, Ref: 5183-2085)
- HPLC Vials, 2 mL (J.G. Finneran, Ref: 32009M-1232)
- HPLC Vial Cap, pre-slit septa (ThermoScientific, Ref: C5000-45B)
- Pursuit 3 PFP 2.0 x 100 mm 3 µm HPLC column (Agilent, Ref: A3051100x020)
- AB Sciex 6500 Mass Spectrometer with Water's I-Class Acquity UPLC with flow-through needle injector
- *(alternatively) A Waters Xevo-TQ-S Mass Spectrometer is sensitive enough to detect GAG markers*

Urinary Creatinine Measurement:
- Creatinine, anhydrous (CAS 60-27-5) (Sigma Aldrich, Ref: C4255)
- d3-Creatinine (CAS 143827-20-7) (Cayman Chemical, Ref: 16763)
- MicroTube Rack System, 0.65 mL polypropylene (Unifit Brand Tips, Ref: TN0933-01R)
- Waters Acquity UPLC BEH Shield RP18 1.7 µm column (Waters, Ref: 186002854)
- Waters Xevo-TQ Mass Spectrometer with Water's Acquity UPLC with fixed loop injector

PATIENT SAMPLE PREP (Fibroblast, Urine, or DBS)

Fibroblast Samples:
Suspend pelleted fibroblasts in 1x PBS and lyse via freeze-thaw in -80°C freezer. Mix well via vortexing, centrifuge, and collect the supernatant in a new tube. Measure the protein content in the supernatant using the Pierce BCA assay kit from ThermoFisher (BSA standard). Transfer 120 µg protein to a labeled 0.5 mL PCR tube and dry in a SpeedVac concentrator. A smaller amount of protein may be used (as low as 80-90 µg), but at least 120 µg is recommended for measurement of MPS-II marker, which is the least abundant of all MPS markers, and unknown samples. Depending on the volume of buffer, drying may take up to 3h (for approximately 200 µL). If the protein content of a cell pellet is already known, a 2.4 mg/mL solution should be made with 1x PBS and the sample should be divided into 50 µL aliquots after vortexing (50 µL contains 120 µg protein).

Urine Samples:
Determine the concentration of urinary creatinine using the LC-MS method described at the end of this SOP. Transfer an aliquot of the urine containing 0.011 µmol creatinine to a labeled 0.5 mL PCR tube and dry in a SpeedVac concentrator. Depending on the dilution of urine, this will likely be a small volume of urine (between 2-25 µL) that will dry quickly.

DBS Samples:
Take two 3 mm punches from the DBS spot and transfer them to a 1.5 mL capped microtube. Alternatively, one 3 mm punch may be used – MPS-I samples were tested successfully in Gelb Lab with a single punch. Add 50 µL MilliQ water, cap, and incubate for 2 h at 37°C while shaking at 250 rpm. Centrifuge for 5 seconds at 1000*g* to collect liquid at the bottom of the tube, and transfer liquid to a labeled 0.5 mL PCR tube. Dry the sample in a SpeedVac.

METHODOLOGY

Prepare the following solutions:

- <u>0.2 mM Chondroitin disaccharide di-4S (IS) stock in water</u>: To 1 mg chondroitin disaccharide di-4S (503.34 g/mol), add 198.7 µL MilliQ water to make 10 mM solution. Dilute 10 µL of the 10 mM stock to 500 µL using MilliQ water to make 0.2 mM stock. Aliquot the 0.2 mM stock into vials with Teflon-septum screw caps and store all stocks at -20°C. Seal the vials with Parafilm to prevent evaporation.
- <u>0.2 M Formic Acid in water</u>: Dilute 3.773 mL Optima Grade formic acid (46.03 g/mol; 1.22 g/cm^3) to 500 mL using MilliQ water.
- <u>0.4 M Ammonia in 24% Methanol in Water</u>: Mix 13.5 mL MilliQ water with 11.9 mL HPLC grade methanol. Add 2.697 mL of 28-30% NH_3. Mix gently to combine and use within 1 week.
- <u>0.25 M PMP with 1.0 µM IS:</u> Weigh out 43.6 mg PMP and dissolve in 1.0 mL of 0.4 M ammonia solution. The solution may be heated up to 70°C to help dissolve PMP solids if necessary. Spike the solution with 5.0 uL of 0.2 mM chondroitin disaccharide di-4S ("IS"). Prepare this solution fresh before each derivatization reaction.

Start the PCR program to warm up the tray (set the program to run at 70°C for 10h or the maximum time allowed so the timer doesn't run out during your experiment). To each dried sample in a 0.5 mL PCR tube, add 100 µL of the 0.25 M PMP solution with 1.0 µM IS. Mix the reaction tubes well via vortexing, then load them onto the PCR tray and start a timer for 90 min.

After 90 min, remove the samples from the PCR tray and allow them to cool for 10 minutes (cancel the remainder of the PCR program). As reaction mixtures cool, they may be transferred to 1.5 mL capped microcentrifuge tubes. After 10 minutes, dilute the samples with 500 µL of 0.2 M formic acid in water to acidify, cap the tube, and vortex. To each acidified sample, add 500 µL chloroform to wash PMP from the aqueous layer, cap the tube, and shake vigorously by hand for one minute. For large numbers of samples, load the tubes into a microcentrifuge tube rack, place another rack on top to hold them in place, and shake all samples together. Centrifuge the samples for 1 minute at 13000g to separate the layers (centrifuge longer if layers are not separated). Pre-wet a disposable Pipetman tip with clean chloroform and use it to remove the bottom chloroform layer of the sample for disposal. Reserve the aqueous layer in the 1.5 mL tube. Wash the aqueous layer three more times (for a total of four times) with 500 µL chloroform, following the steps above.

When the chloroform washes are complete, concentrate the aqueous layer in a SpeedVac concentrator to a volume of approximately 70 µL. Centrifuge the samples for 5 minutes at 13,000*g* to pellet any solids. Being careful not to disturb the bottom,

transfer the aqueous sample to a 2 mL HPLC vial containing a 250 µL pulled-point glass insert for analysis via LC-MS as described below.

LC-MSMS Analysis:

The derivatized glycosaminoglycan (GAG) markers were separated using a 2.0x100 mm 3 µm Pursuit 3 PFP column (Agilent P/N: A3051100X020) connected to a Waters I-Class Acquity UPLC with a direct infusion syringe pump. The column was held at room temperature and the markers were eluted with a 12-minute UPLC gradient program using 0.1% formic acid in water (MPA) and 0.1% formic acid in acetonitrile (MPB). A strong needle wash of 100% acetonitrile and a purge wash and seal wash of 100% water were used. Markers were detected with an AB Sciex 6500 TQ mass spectrometer with an IonSpray source in negative mode. Nitrogen was used as a curtain gas at 30 psi, collision gas was 12 units, IonSpray voltage was -4500 V, source temperature was 400°C, and ion source gas 1 and gas 2 were each 40 units. See tables below for UPLC gradient program, MRM tuning information, and marker retention times.

UPLC Solvent Gradient Program:

Time (min)	Flow (mL/min)	%MPA	%MPB
0.0	0.3	85	15
5.0	0.3	77.5	22.5
5.5	0.3	10	90
7.5	0.3	10	90
8.0	0.3	85	15
12.0	0.3	85	15

Marker Names, Retention Times, MRMs, and Tuning Information for AB Sciex 6500

Marker	MPS Type	Retention Time (RT)	MRM	DP	EP	CE	CXP
UA-GalNAc-4S	Internal Standard	3.69 min	788.1 > 534.1	-70	-10	-35	-25
HNAc (1S)	MPS-IVA	4.42 min	630.4 > 256.1	-80	-10	-45	-25
HNAc (2S) *	MPS-VI	n/a	710.2 > 256.1	-80	-10	-45	-25
HNAc-UA (1S) †	MPS-IVA	4.67 min	806.0 > 331.1	-84	-10	-45	-25
HN-UA (1S)	MPS-IIIA	4.40 min	764.2 > 331.1	-80	-10	-45	-25
UA-HNAc (1S) (early RT)	MPS-I	3.75 min	806.3 > 294.9	-84	-10	-45	-25
UA-HNAc (1S) (late RT)	MPS-II	4.07 min	806.3 > 294.9	-84	-10	-45	-25
UA-HN-UA (1S)	MPS-VII	4.18 min	940.0 > 331.1	-80	-10	-44	-25
$(HNAc-UA)_2$ (1S) ‡	MPS-IIIB	4.00 min	1185.2 > 931.4	-80	-10	-45	-25
$(HNAc-UA)_2$ (2S)	MPS-IIID	4.11 min	632.3 > 298.0	-80	-10	-45	-25
$(HN-UA)_2$-HNAc (2S)	MPS-IIIC	3.69 min	691.8 > 605.0	-40	-10	-24	-25
$(Hex-HNAc)_2$ (2S)	MPS-IVB	3.29 min	1240.0 > 256.1	-87	-10	-41	-25

* Marker peak was not present at MRM reported by Saville et. al.
† Marker peak was present in urine control, but not fibroblast control or DBS patient
‡ Marker peak was present in urine control and fibroblast control, but not in DBS patient

(Alternative Option) Xevo TQ-S Parameters

Parameter	Setting
Polarity	ESI-
Capillary (kV)	2.0
Source Temperature (°C)	150
Desolvation Temperature (°C)	600
Cone Gas Flow (L/hr)	150
Desolvation Gas Flow (L/hr)	900
Collision Gas Flow (mL/min)	ON
Collision Gas	Argon

(Alternative Option) Marker Names, Retention Times, MRMs, and Tuning Information for Xevo TQ-S

Marker	MPS Type	Retention Time (RT)	MRM	Cone (V)	Collision
UA-GalNAc-4S	Internal Standard	4.75 min	788.1 > 534.1	42	24
HNAc (1S)	MPS-IVA	5.55 min	630.4 > 256.1	24	28
HNAc-UA (1S)	MPS-IVA	5.78 min	806.0 > 331.1	28	28
HN-UA (1S)	MPS-IIIA	5.53 min	764.2 > 331.1	32	26
UA-HNAc (1S) (early RT)	MPS-I	4.88 min	806.3 > 294.9	40	27
UA-HNAc (1S) (late RT)	MPS-II	5.23 min	806.3 > 294.9	40	27
UA-HN-UA (1S)	MPS-VII	5.33 min	940.0 > 331.1	44	34
$(HNAc-UA)_2$ (1S)	MPS-IIIB	5.15 min	1185.2 > 931.4	30	32
$(HNAc-UA)_2$ (2S)	MPS-IIID	5.20 min	632.3 > 298.0	30	34
$(HN-UA)_2$-HNAc (2S)	MPS-IIIC	4.80 min	691.8 > 605.0	28	14
$(Hex-HNAc)_2$ (2S)	MPS-IVB	4.35 min	1240.0 > 256.1	28	36

LC-MSMS MEASUREMENT OF URINARY CREATININE

Calculations for urine dilution were made based on the redwoodtoxicology.com claim that average urinary creatinine levels range from 40-300 mg/dL. This translates to concentrations of 3.5-26.5 mM creatinine in an undiluted urine sample (113.12 g/mol creatinine). A 2500x dilution of undiluted urine samples would yield a possible range of 1.4-10.6 µM creatinine (an acceptable range for measurement via LC-MS).

Dilute liquid urine samples 5x in MilliQ water.

Urine Filter Paper Extraction (*ref: Miki K, Sudo A. 1998. Effect of urine pH, storage time, and temperature on stability of catecholamines, cortisol, and creatinine. Clin. Chem. 44(8): 1759-1762.*):

If urine is provided dried on filter paper, extract the urine using 5x the volume of urine originally saturated in the paper. The amount of volume of water to use may be determined by taking the weight of the urine filter paper and dividing the total volume of urine blotted by the weight of the paper. A fraction of the paper may be cut off, weighed, and the weight of the fraction should be multiplied by the urine volume/paper weight value to determine the original volume of urine saturated in the filter paper. Multiply this number by five to determine the amount of water to use in urine extraction. Store the remaining filter paper under desiccant at -20°C.

$$\frac{Total\ Filter\ Paper\ Weight}{Saturated\ Urine\ Volume} \times Weight\ of\ Filter\ Paper\ Segment \times 5 = Water\ for\ Urine\ Extraction$$

Place the filter paper fragment into a 50 mL falcon tube. If the fragment is too large to be completely submerged in the predetermined volume of water (5x original saturated volume), cut it into smaller pieces. Add the water and mix via vortexing. Allow the samples to sit at 4°C overnight and mix again via vortexing the next morning. Briefly centrifuge the samples (5 seconds at 1,000*g*) to collect the liquid at the bottom of the tube. Remove the liquid using a 1,000 mL pipette, pressing the paper with the pipette tip to squeeze out as much liquid as possible. Transfer the liquid to a labelled container such as a 15 mL falcon tube or a 1.5 mL microcentrifuge tube for storage at -20°C.

From the 5x diluted liquid urine or 5x diluted urine extract, take 2 µL and bring to a volume of 1.0 mL using MilliQ water to make 2,500x diluted urine samples. Prepare a serially diluted scaffold of standards for linear regression and measurement of creatinine concentration following the procedure below.

Starting with a 20 mM solution of creatinine in water, prepare a set of nine creatinine standards ranging from 250 nM to 15 µM in water. These standards are used to measure the concentration of 2500x diluted urine samples with a formula obtained by linear regression. The concentrations to prepare are 15 µM, 12 µM, 8 µM, 5 µM, 2 µM, 1 µM, 750 nM, 500 nM, and 250 nM.

LC-MS Analysis For Creatinine Measurement in Urine:

Urinary creatinine was measured using a Waters Acquity UPLC with fixed loop injector connected to a Waters Xevo TQ mass spectrometer. Compounds were separated with a Waters Acquity UPLC BEH Shield RP18 1.7 µm 2.1x100 mm column (Waters P/N 186002854) using a 3 minute isocratic method of 50% 0.1% formic acid in water (MPA) and 50% 0.1% formic acid in acetonitrile at a flow rate of 0.2 mL/min. The weak needle wash was 0.1% formic acid in 90/10 water/acetonitrile, the strong needle wash was MPB, and the seal wash was 50/50 water/acetonitrile.

CREATININE MRM/MS TUNE

Analyte	Precursor (m/z)	Product (m/z)	Cone (V)	Collision (V)
Creatinine	114.1	44	40	15
Creatinine-d3	117.1	89	40	15

WATERS XEVO TQ SETTINGS

Parameter	Setting
Polarity	ESI+
Capillary (kV)	3.50
Cone (V)	25.00
Extractor (V)	3.00
Source Temperature (°C)	150
Desolvation Temperature (°C)	400
Cone Gas Flow (L/Hr)	50
Desolvation Gas Flow (L/Hr)	500
Collision Gas Flow (mL/min)	0.15
LM 1 Resolution	3.0
HM 1 Resolution	15.0
LM 2 Resolution	2.8
HM 2 Resolution	15.0

Version: 07-15-2020

SOP FOR DETERMINATION OF GAG LEVELS IN MPS-I PATIENT SAMPLES VIA PROTEASE DIGESTION, PURIFICATION, ENZYME DIGESTION, AND GRIL-LC/MS (SENSI-PRO FULL METHOD)

MATERIALS

Samples:

- Donated anonymous healthy newborn dried blood spots (DBS) (from Dr. Francyne Kubaski, Universidade Federal do Rio Grande, Brazil and Dr. Zoltan Lukacs, University of Hamburg, Germany)
- Donated anonymous MPS-I newborn dried blood spots (DBS) (from the National MPS Society, United States)

Standards and Reagents:

- Chondroitin/Dermatan Sulfate Non-Reducing End (NRE) "I0S0" (From BioMarin)
- Chondroitin/Dermatan Sulfate Non-Reducing End (NRE) "I0S6" (From BioMarin)
- Heparan Sulfate Disaccharide "D0A0" (CAS: 136098-07-2) (Iduron, Ref: HD006)
- Heparan Sulfate Disaccharide "D0S0" (CAS: 136098-08-3) (Iduron, Ref: HD005)
- Pronase® from *Streptomyces griseus* (CAS 9036-06-0) (Sigma Aldrich, Ref: P5147)

- Sodium Acetate Trihydrate (CAS 6131-90-4) (Ward's Science, Ref: 470302-428)
- Sodium chloride (CAS 7440-23-5) (Sigma Aldrich, Ref: S9888)
- 190-Proof Ethanol (CAS 64-17-5) (Fisher, Ref: 22-032-600)
- Sodium azide (CAS 26628-22-8) (Sigma Aldrich, Ref: S2002)
- Heparinase I from *Flavobacterium heparinum*, 0.5 IU, lyophilized (Ibex, Ref: 60-400, 1IU* = 1.0 µmole/min)
- Heparinase II from *Flavobacterium heparinum*, 0.5 IU, lyophilized (Ibex, Ref: 60-400, 1IU* = 1.0 µmole/min)
- Heparinase III from *Flavobacterium heparinum*, 0.5 IU, lyophilized (Ibex, Ref: 60-400, 1IU* = 1.0 µmole/min)
- Bovine Serum Albumin (BSA) lyophilized powder (Sigma, Ref: A0281-10G)
- HEPES Buffer Salt (CAS 7365-45-9) (Fisher, Ref: BP-310-500)
- Calcium chloride (CAS 7440-70-2) (Sigma Aldrich, Ref: C-3306)
- Dithiothreitol (DTT) (CAS 3483-12-3) (Sigma Aldrich, Ref: D0632)
- [$^{12}C_6$] Aniline (CAS 62-53-3) (Sigma Aldrich, Ref: 242284)
- [$^{13}C_6$] Aniline (CAS 100849-37-4) (Sigma Aldrich, Ref: 485497)
- Sodium Cyanoborohydride (CAS 25894-60-7) (Oakwood Chemical, Ref: 044871)
- Dimethyl Sulfoxide, DMSO (CAS 67-68-5) (Fisher, Ref: BP231-100)
- Glacial Acetic acid (CAS 64-19-7) (Fisher, Ref: A38S-500)
- Dibutylamine (CAS 111-92-2) (Sigma Aldrich, Ref: 471232)
- Methanol, HPLC Grade (CAS 67-56-1) (Fisher, Ref: A452-1)
- Ammonia Solution 28-30% (CAS 1336-21-6) (MilliporeSigma Ref: 105423)
- Methanol, Optima Grade (CAS 67-56-1) (Fisher, Ref: A456-4)
- Water, Optima Grade (CAS 7732-18-5) (Fisher, Ref: W6500)

Benchtop Supplies:
- 1.5 mL Microcentrifuge tube with snap cap (Fisher, Ref: 05-408-129)
- pH Test strips, 4.5-10.0 pH (MilliporeSigma, Ref: P4536)
- DEAE Sephacel (GE Healthcare, Ref: 17-0500-01)
- Nunc™ 96-well Filter Plate, 20 µm pore frit (Thermo, Ref: 278011)
- Deep well 96-well plate, 2.2 mL, square well, polypropylene (Thermo, Ref: AB0932)
- PD-10 Desalting Columns (GE Healthcare, Ref: 17-0851-01)
- 4 mL Collection Tubes (VWR, Ref: 60818-096)
- 2 mL Microcentrifuge Tubes with snap caps (Thomas Scientific, Ref: 1159V26)
- MilliQ Water Filtration System
- Heating Block (37°C) in Fume Hood
- Incubator set to 37°C with Shaker
- Allegra X-12R Centrifuge (Beckman Coulter)
- SpeedVac, Vacuum Concentrator
- Benchtop Centrifuge

Mass Spectrometry Supplies/Equipment:
- Pulled-point glass HPLC vial inserts, 250 µL (Agilent, Ref: 5183-2085)
- HPLC Vials, 2 mL (J.G. Finneran, Ref: 32009M-1232)
- HPLC Vial Cap, pre-slit septa (ThermoScientific, Ref: C5000-45B)
- Acquity UPLC Column BEH C18 1.7µm 2.1x100mm Column (Waters, Ref: 186002352)
- Acquity UPLC BEH C18 1.7µm 2.1x5mm Pre-Column (Waters, Ref: 186003975)
- Waters Xevo TQ-S Mass Spectrometer with Water's I-Class Acquity UPLC with flow-through needle injector

METHODOLOGY

Prepare the following solutions:

- Pronase® Working Solution in MilliQ Water (20 mg/mL). Dilute 10 mg of Pronase® with 500 µL MilliQ Water. Aliquot and store solution in Teflon-septum capped glass vials sealed with Parafilm at -20°C for up to one year.
- 0.24M sodium acetate trihydrate, 1.92 M NaCl, pH 6.5. Measure 163 mg of sodium acetate trihydrate and 561 mg of sodium chloride into a 15 mL falcon tube. Dissolve in 5 mL MilliQ water. Check the pH with pH strips.
- 1 mg/mL Pronase® in 0.24M sodium acetate trihydrate, 1.92 M NaCl. Dilute 240 µL of 20 mg/mL Pronase® solution to 4600 µL with 0.24M sodium acetate trihydrate, 1.92 M NaCl, pH 6.5. Mix gently with a pipette and use immediately.
- DEAE Equilibration Buffer: 20 mM sodium acetate trihydrate 200 mM NaCl, pH 6.0. Measure 1632 mg sodium acetate trihydrate and 7012 mg NaCl into a 1 L jar. Dissolve in 600 mL of MilliQ Water. Store at 4°C until needed. Bring to room temperature prior to use. Check pH with pH strips.
- DEAE Elution Buffer: 20 mM sodium acetate trihydrate 1200 mM NaCl, pH 6.0. Measure 272 mg sodium acetate trihydrate and 7013 mg NaCl into a 500 mL jar. Dissolve in 100mL of MilliQ Water. Store at 4°C until needed. Bring to room temperature prior to use. Check pH with pH strips.
- 10% Ethanol in MilliQ Water. Prepared by diluting 75 mL ethanol (200-proof) to 750 mL with milliQ water.
- 0.01% Sodium Azide. Measure 17 mg sodium azide into a 250 mL glass jar. Dilute to 170 mL with MilliQ water.
- 1% BSA in water: Dissolve 0.5 g BSA in 50 mL MilliQ water. Store at 4°C.
- 1 mg/mL I0S0 in water: Make 1 mg/mL stock by adding 1 mL MilliQ water to 1 mg of I0S0 powder. Store in Teflon-septum capped glass vials sealed with Parafilm at -20°C.
- 1 mg/mL I0S6 in water: Make 1 mg/mL stock by adding 1 mL MilliQ water to 1 mg of I0S6 powder. Store in Teflon-septum capped glass vials sealed with Parafilm at -20°C.
- 2.5 µg I0S0 and I0S6 Aliquots for Aniline labelling: Since only10 µg I0S0 and 10 µg of I0S6 NREs were received from Biomarin, these NREs were reconstituted in 100 µL water and then aliquoted into four 25 µL fractions in labeled 1.5 mL microcentrifuge tubes. The NRE solutions were dried via Speed Vac at room temperature and the aliquots were stored at -80°C until use.
- 1 mg/mL (2.49 mM) D0A0 in water: Make 1 mg/mL stock by adding 1 mL MilliQ water to 1 mg of D0A0 powder. Store in Teflon-septum capped glass vials sealed with Parafilm at -20°C.
- 1 mg/mL (2.17 mM) D0S0 in water: Make 1 mg/mL stock by adding 1 mL MilliQ water to 1 mg of D0S0 powder. Store in Teflon-septum capped glass vials sealed with Parafilm at -20°C.
- 50 µg D0A0, and D0S0 Aliquots for Aniline labelling: Prepare the 1 mg/mL internal standard stocks (D0A0 and D0S0) divide into 50 µL aliquots in labeled 1.5 mL microcentrifuge tubes. Dry down the solutions via Speed Vac at room temperature and store the aliquots at -80°C until use.
- 20 mM HEPES-HCl, 3.3 mM CaCl2, pH 7.0. Measure 142.8 mg HEPES and 11 mg of CaCl2 into a 50 mL falcon tube. Add 15 mL H2O and dissolve. Adjust pH with NaOH and HCl to 7.0. Bring volume to 30 mL.
- 100 mM DTT Solution. Dissolvµe 15 mg DTT per 1.00 mL of MilliQ Water.
- Heparinase I, II, and III stocks (2.5 IU/mL)*: Dissolve Heparinase I, II, and III (0.5 IU) in 0.2 mL of 1% BSA in water. This is a final concentration of 2.5 IU/mL. Aliquot into 10 µL fractions and store at -20°C to avoid multiple freeze-thaw cycles. More dilute solutions may be made if less than 20 samples will be run at a time.
- Heparinases Mixture (100 mIU/mL). Combine 20 µL of each Heparinase I, II, and III enzyme solution (2.5 IU/mL) and dilute to 500 µL with 1% BSA.
- 1 M Sodium Cyanoborohydride in DMSO:Acetic Acid (7:3, v/v): For ~50 samples, weigh 58.35 mg of sodium cyanoborohydride into a 4 mL glass vial and dissolve in 649.7 µL of DMSO and 278.8 µL Glacial Acetic Acid. Vortex the vial and allow to sit for 30 min, then vortex again. Allow the vial and contents to sit at room temperature for an additional 90 minutes (total time of 2h) to ensure sodium cyanoborohydride is completely dissolved.
- Mobile Phase A (MPA), 8mM Acetic Acid, 5 mM dibutylamine (DBA) in 100% water. To prepare 500 mL of MPA, add 228.8 µL glacial acetic acid and 421.3 µL DBA to 500 mL Optima grade water. Mix well.
- Mobile Phase B (MPB), 8mM Acetic Acid, 5 mM dibutylamine (DBA) in 30:70 water:methanol. To prepare 500 mL of MPB, add 228.8 µL glacial acetic acid and 421.3 µL DBA to 150 mL Optima grade water. Dilute with 350 mL of Optima grade methanol. Mix well.

** Enzymes seem to be stable with up to 3 freeze-thaw cycles, so make aliquots such that you can keep the number of cycles to 3, discarding after 3 cycles.*

Day 1 : Pronase® Digestion
Day 2 : DEAE Chromatography and PD-10 Desalting
Day 3 : Heparinase Digestion
Day 4 : Aniline tagging

Day 5 : Dry on SpeedVac
Day 6 : LC-MS/MS injection

Pronase® Digestion of DBS Samples:
Punch one 3 mm DBS disk per patient and put the punches into a 1.5 mL microcentrifuge tube with 50 µL MilliQ water. Incubate at 37°C while shaking at 250 rpm for 2h. Briefly centrifuge to collect all liquid at the bottom of the tube, then carefully add 100 µL of 1 mg/mL Pronase® working solution and mix carefully with the pipette. Incubate for 20 h at 37°C without shaking.

Purification by Anion Exchange Chromatography:
After 24 h, dilute Pronase® samples with 850 µL of water (for a final NaCl concentration of 192 mM, final NaAcetate concentration 24 mM, and final volume of 1 mL) and mix via vortexing.

Approximately 2h prior to GAG purification, measure the DEAE Sephacel into a 50:50 DEAE:Water (v/v) slurry and transfer to a 50 mL wide-mouth bottle to mix with a magnetic stir bar (25 mL total prepared). Place a 96-well fritted filter plate on top of a 2.2 mL 96-well plate and add 100 µL of water to each well followed by 500 µL of the 50:50 DEAE:Water slurry. Centrifuge the fritted filter plate for 1 minute at 800 *g* to remove water. A volume of 250 µL of DEAE Sephacel should remain in the filter plate.

Remove the eluate from each well of the collector plate and equilibrate the resin with 7mL of Equilibration Buffer in 1 mL increments, centrifuging for 1 min at 800 *g* between each increment. Dispose of eluate in waste. After equilibration, pipet the 1 mL Pronase® samples into their respective well in the filter plate. Centrifuge the plate for 1 min at 800 *g*, and dispose of eluate in waste. Wash the resin with an additional 5 mL of Equilibration Buffer in 1 mL increments and centrifuge for 1 min at 800 *g* between each increment. Dispose of eluate in waste.

Finally, add a total of 2 mL of Elution Buffer to the resin in 1 mL increments. Centrifuge the plate for 1 m at 800 *g* between each increment and collect the filtrate and save for the desalting step. Below is a summary of the anion exchange chromatography equilibration and elution process:

1 mL (7x) Equilibration Buffer (collect and dispose of eluate)
1 mL (1x) DBS/Pronase® sample (collect and dispose of eluate)
1 mL (5x) Equilibration buffer (collect and dispose of eluate)
1 mL (2x) Elution Buffer (collect and save separately)

Desalting with PD-10 Columns:
Make sure that PD-10 columns are at room temperature by allowing them to equilibrate on the benchtop for at least 15 minutes prior to use. Note that these columns may be re-used up to 10 times. Mark each column every time it is used and dispose of it after ten uses.

Decant the storage liquid from the column. Equilibrate the column with 12 mL of 10% Ethanol in MilliQ Water. Discard eluate into waste. For each sample, pipette the 2 mL of DEAE purified sample to a PD-10 column and add an additional 0.5 mL of 10% Ethanol once the sample is completely loaded. Collect this eluate to save in case of loss of molecules of interest or send it to waste. Elute the disaccharides and NREs with 3.5 mL of 10% Ethanol in MilliQ water and collect the eluate in clean 4 mL VWR tubes. A summary of this process is shown below.

12 mL 10% Ethanol in water (collect and dispose of eluate)
2 mL of DEAE sample (collect and dispose of eluate)
0.5 mL 10% Ethanol in water (collect and dispose of eluate)
3.5 mL 10% Ethanol in water (collect in 4 mL tubes and save)

Dry the eluate (3.5 mL) in SpeedVac overnight (16-18h) at room temperature. Re-condition the column by washing with 20 mL of MilliQ water, then wash with 2.5 mL of 0.01% sodium azide solution. Place stoppers in the bottom of each column, add 2.5 mL of 0.01% sodium azide solution, and replace the top cover. Mark the column with the number of usages, discarding columns that have been used 10 times. Store columns at 2-8°C.

Digestion of GAGs with Heparinases:

Reconstitute the desalted GAGs into 50 µL water, vortexing to dissolve, and centrifuging to bring all liquid to the bottom of the tube, then incubate for 30 min at room temperature to ensure all GAGs have dissolved.

Label a 2.0 mL microcentrifuge tube for each sample and pipette the reconstituted samples into their respective tube. Dry the samples via SpeedVac.

Prepare the enzyme incubation mixture (for 50 samples) by combining 2.5 mL of HEPES buffer and 150 µL of 100 mM DTT solution, mix via vortexing. Then, add 500 µL of heparinases and mix gently with pipette. Add 63 µL of this solution to each 2.0 mL microcentrifuge tube (50 µL HEPES, 3 µL DTT, 10 µL Heparinases). Close the cap on each tube and incubate at 37°C, shaking at 250 rpm, for 16 h.

Aniline Labelling of NREs and Internal Disaccharides by Reductive Amination:
After 16 h of incubating, dry the heparinase reaction mixture via SpeedVac. Begin preparing sodium cyanoborohydride reagent in 7:3 DMSO:acetic acid (v/v) 2 h in advance of reaction start time.

Calibrators, controls, and samples are tagged with [$^{12}C_6$] aniline. Internal Standards (ISTDs) are tagged with [$^{13}C_6$] aniline. Tagging must be performed under the hood. Layer the aniline with nitrogen and protect from light before storage. Set the heat block to 37°C (±2°C).

Add 15 µL of [$^{12}C_6$] aniline and 15 µL of 1M NaCNBH3 to each dry heparinase-digested sample in a 2 mL microcentrifuge tube, then vortex the samples. Add 15 µL of [$^{13}C_6$] aniline and 15 µL of 1M NaCNBH3 to one vial of each dry I0S0 and I0S6 NRE (2.5 µg) and to one vial of each dry D0S0 and D0A0 disaccharide (50 µg), then vortex the samples. Incubate samples and standards at 37°C for 30 minutes, then vortex again. Leave samples and standards to react at 37°C for an additional 15.5h (16h total) without shaking.

After 16h reacting, remove the tubes from the heat and dry via SpeedVac at room temperature for at least 24h.

Preparation for LC-MS/MS Analysis:
Reconstitute the dried [$^{13}C_6$] aniline labelled NRE ISTDs (I0S0 and I0S6) with 200 µL of Mobile Phase A to make 12.5 ng/µL and vortex. Reconstitute the dried [$^{13}C_6$] aniline labelled disaccharide ISTDs (D0S0 and D0A0) with 500 µL of Mobile Phase A to make 100 ng/µL solutions and vortex. Allow the dried ISTDs to incubate at room temperature for 30 minutes before vortexing again, then centrifuging for 15 min at 3,000 *g* to pellet particulates. Store the reconstituted ISTD solutions in labelled glass vials with Teflon-septum screw caps, sealed with Parafilm, at -20°C if not used immediately.

In order to reconstitute 50 dried [$^{12}C_6$] aniline labelled samples, prepare a 5 mL solution of 125 ng/mL I0S0 and I0S6 NRE ISTDs in MPA by combining 50 µL of I0S0 12.5 ng/µL stock with 50 µL of I0S6 12.5 ng/µL stock and diluting to 5 mL with MPA. D0A0 and D0S0 ISTDs could also be added to the 5 mL reconstitution solution if desired. Mix the 5 mL reconstitution solution well, then reconstitute dried [$^{12}C_6$] aniline labelled samples by adding 100 µL of the 125 ng/mL I0S0 and I0S6 in MPA stock. Vortex each sample and allow to incubate at room temperature for 30 minutes before vortexing a second time and centrifuging for 15 min at 3,000 *g*. Without disturbing the pellet, carefully transfer the liquid to a 2 mL HPLC vial containing a 250 µL pulled-point glass insert.

LC-MS/MS Analysis:

Aniline-labelled NREs and internal disaccharides were separated using an Acquity UPLC Column BEH C18 1.7µm 2.1x100mm Column (Waters, Ref: 186002352) fitted with an Acquity UPLC BEH C18 1.7µm 2.1x5mm Pre-Column (Waters, Ref: 186003975) connected to a Waters I-Class Aquity UPLC with a flow-through needle. The column was held at 40°C. The markers were eluted in a 15-minute UPLC gradient program with 8mM acetic acid, 5 mM dibutylamine (DBA) in water (MPA) and 8mM acetic acid, 5 mM dibutylamine (DBA) in 30:70 water:methanol (v/v) (MPB). See the program in the table below. A strong needle wash of 100% acetonitrile, a purge wash of MPA, and a seal wash of 90/10 water/acetonitrile were used.

UPLC Solvent Gradient Program:

Time (min)	Flow (mL/min)	%MPA	%MPB
0.00	0.35	99.0	1.0
2.00	0.35	99.0	1.0
11.00	0.35	0.2	99.8
12.00	0.35	0.2	99.8
13.00	0.35	99.0	1.0
15.00	0.35	99.0	1.0

Disaccharides were detected with a Waters Xevo TQ-S mass spectrometer with an ESI source in negative mode. See mass spec parameters and MRMs below. Waters MassLynx Software and TargetLynx was used to analyze the data.

Xevo TQ-S Parameters

Parameter	Setting
Polarity	ESI-
Capillary (kV)	2.0
Source Temperature (°C)	150
Desolvation Temperature (°C)	600
Cone Gas Flow (L/hr)	150
Desolvation Gas Flow (L/hr)	900
Collision Gas Flow (mL/min)	ON
Collision Gas	Argon

Marker Names, Retention Times, MRMs, and Tuning Information for Xevo TQ-S

Analyte	Precursor	Product	Cone	Collision	
IoSo - [$^{12}C_6$]	510.94	430.81	36	20	
IoSo - [$^{13}C_6$]	516.55	436.63	34	23	
IoS6 - [$^{12}C_6$]*	591.07	431.01	44	32	
IoS6 - [$^{13}C_6$]*	597.03	436.77	50	34	
DoAo - [$^{12}C_6$]	455	157	35	20	MRM From ARUP Protocol
DoAo - [$^{13}C_6$]**	461	157	35	20	MRM From ARUP Protocol
DoSo - [$^{12}C_6$]	493	377	35	20	MRM From ARUP Protocol
DoSo - [$^{13}C_6$]**	499	383	35	20	MRM From ARUP Protocol

** IoS6 peak is can also be seen in the IoSo MRM channel. This is likely due to desulfation of the ion in the source. Due to high background and low IoS6 signal in the IoS6 MRM channel, the IoS6 peaks were processed through the IoSo MRM channel in order to achieve a more accurate peak integration. See example chromatograms.*

*** DoAo and DoSo - [$^{13}C_6$] ISTDs were not run with each assay because these markers can be seen using the Kubaski Method. DoAo and DoSo values were calculated relative to the IoSo - [$^{13}C_6$] ISTD peak for reference.*

EXAMPLE CHROMATOGRAMS:

Below: IoS6 - [$^{12}C_6$] Peak at 591.07 > 431.01, showing some background interference for some peaks.

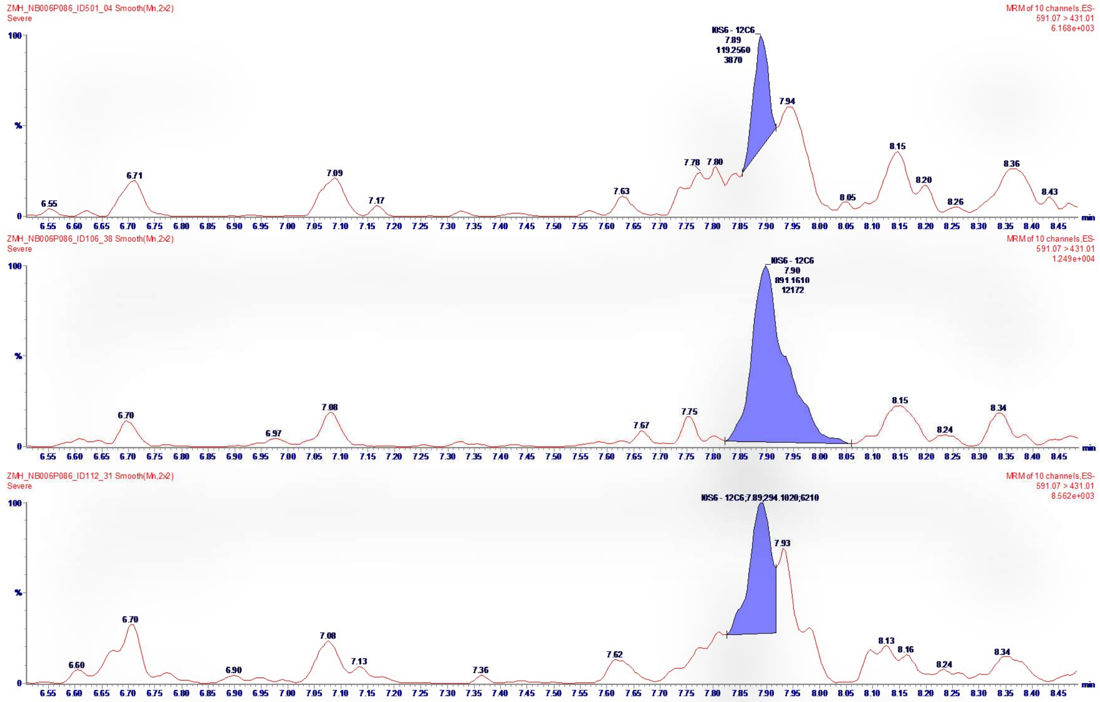

Marker: I0S6 (I0S0 trace) MPS-I (ID: 106) Newborn DBS

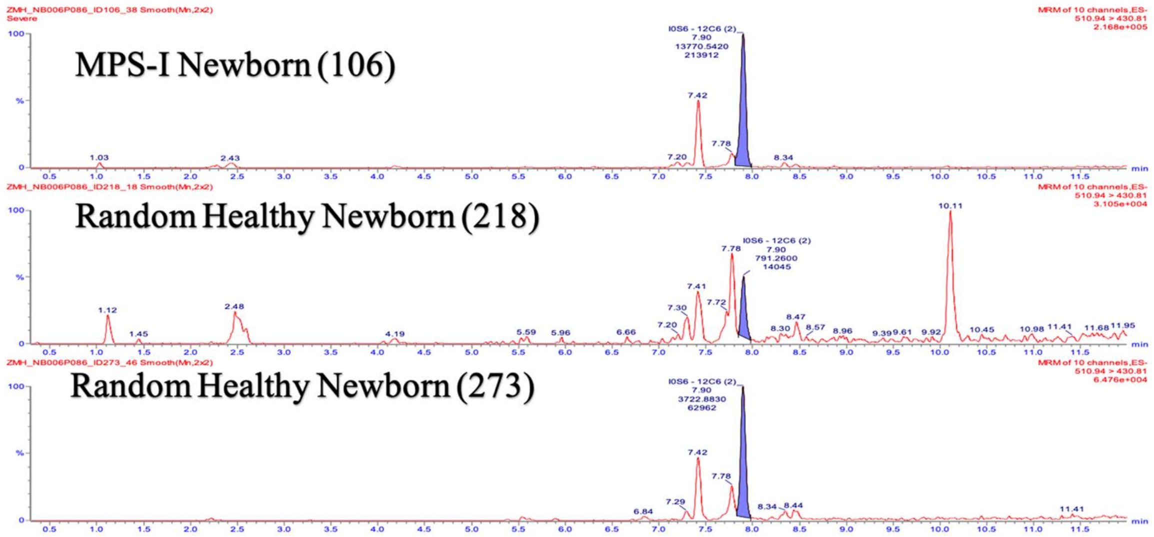

Supplementary Material: SensiPro Full Method SOP

Marker: D0S0 MPS-I (ID: 106) Newborn DBS

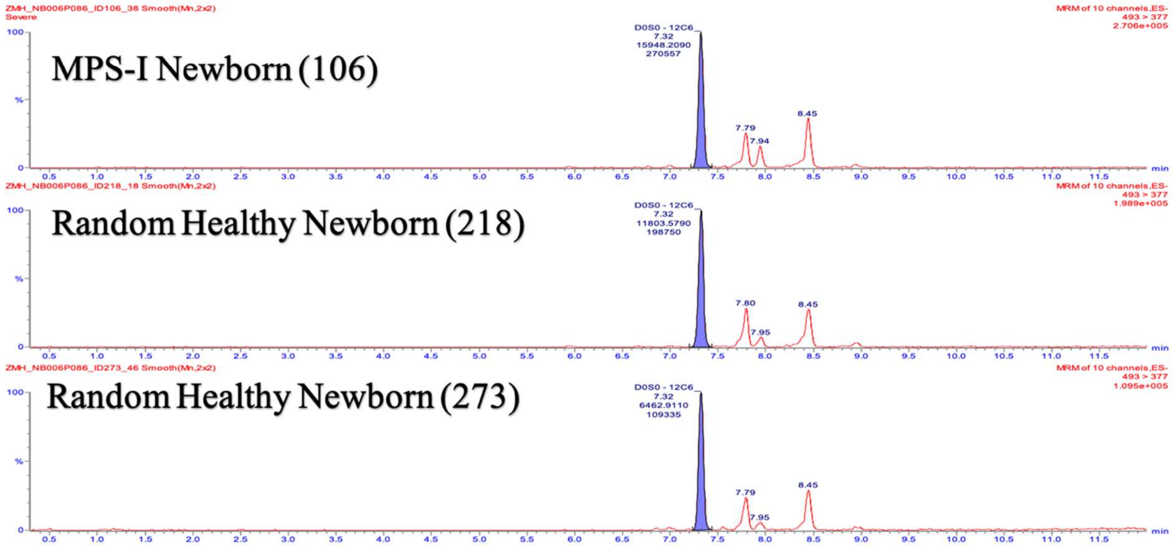

Marker: D0A0 MPS-I (ID: 106) Newborn DBS

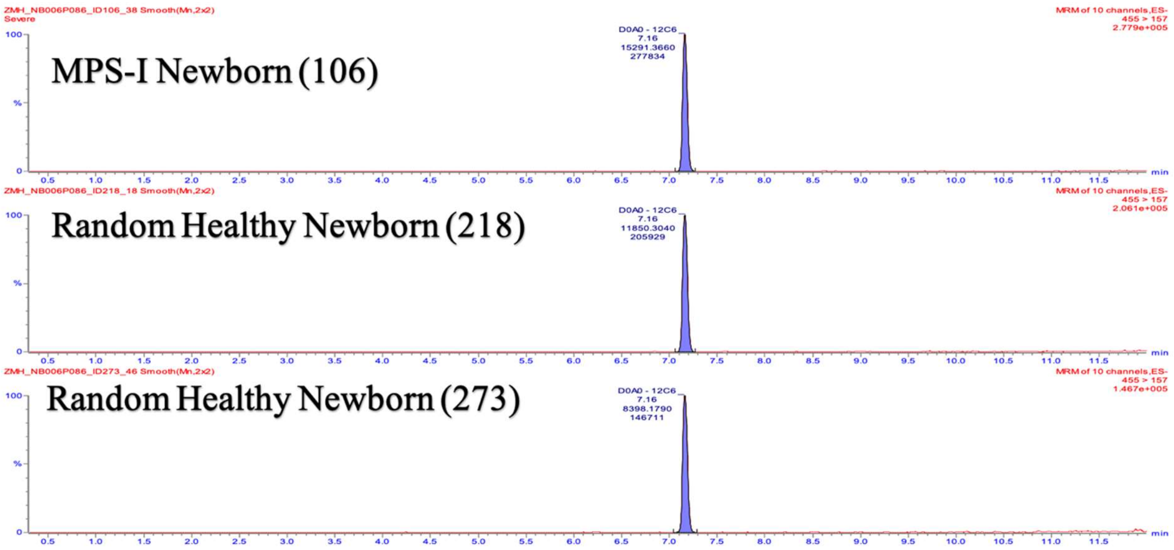

Supplementary Material: SensiPro Full Method SOP

Marker: I0S0 MPS-I (ID: 106) Newborn DBS

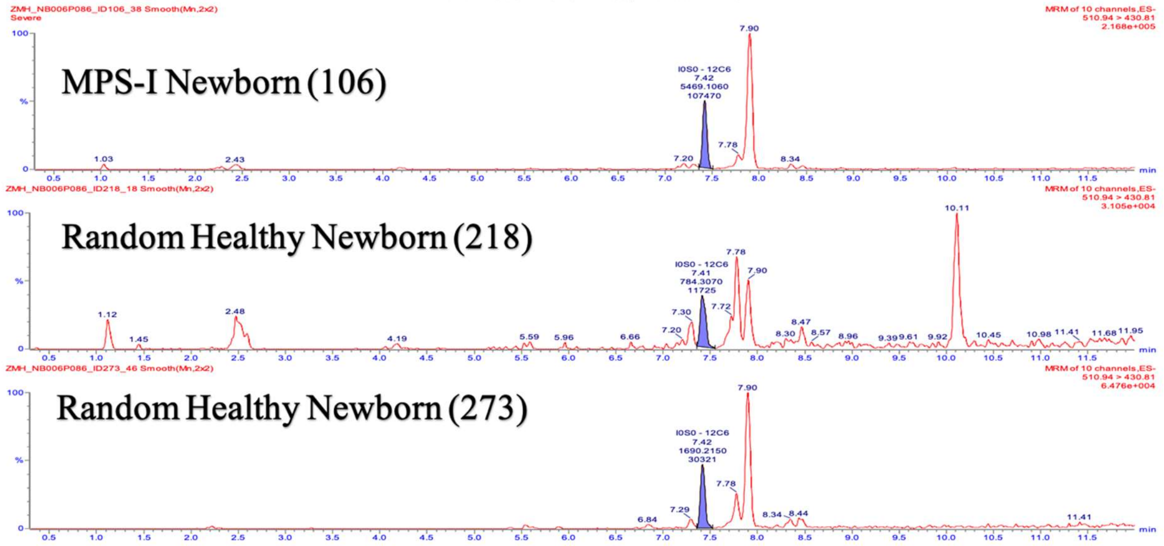

Version: 07-15-2020

SOP FOR DETERMINATION OF GAG LEVELS IN MPS-I PATIENT SAMPLES VIA ENZYME DIGESTION AND GRIL-LC/MS (SENSIPRO LITE METHOD)

MATERIALS

Samples:

- Donated anonymous healthy newborn dried blood spots (DBS) (from Dr. Francyne Kubaski, Universidade Federal do Rio Grande, Brazil and Dr. Zoltan Lukacs, University of Hamburg, Germany)
- Donated anonymous MPS-I newborn dried blood spots (DBS) (from the National MPS Society, United States)

Standards and Reagents:

- Chondroitin/Dermatan Sulfate Non-Reducing End (NRE) "I0S0" (From BioMarin)
- Chondroitin/Dermatan Sulfate Non-Reducing End (NRE) "I0S6" (From BioMarin)
- Heparan Sulfate Disaccharide "D0A0" (CAS: 136098-07-2) (Iduron, Ref: HD006)
- Heparan Sulfate Disaccharide "D0S0" (CAS: 136098-08-3) (Iduron, Ref: HD005)
- Heparinase I from *Flavobacterium heparinum*, 0.5 IU, lyophilized (Ibex, Ref: 60-400, 1IU* = 1.0 µmole/min)
- Heparinase II from *Flavobacterium heparinum*, 0.5 IU, lyophilized (Ibex, Ref: 60-400, 1IU* = 1.0 µmole/min)
- Heparinase III from *Flavobacterium heparinum*, 0.5 IU, lyophilized (Ibex, Ref: 60-400, 1IU* = 1.0 µmole/min)
- Bovine Serum Albumin (BSA) lyophilized powder (Sigma, Ref: A0281-10G)
- HEPES Buffer Salt (CAS 7365-45-9) (Fisher, Ref: BP-310-500)
- Calcium chloride (CAS 7440-70-2) (Sigma Aldrich, Ref: C-3306)
- Dithiothreitol (DTT) (CAS 3483-12-3) (Sigma Aldrich, Ref: D0632)
- [$^{12}C_6$] Aniline (CAS 62-53-3) (Sigma Aldrich, Ref: 242284)
- [$^{13}C_6$] Aniline (CAS 100849-37-4) (Sigma Aldrich, Ref: 485497)
- Sodium Cyanoborohydride (CAS 25894-60-7) (Oakwood Chemical, Ref: 044871)
- Dimethyl Sulfoxide, DMSO (CAS 67-68-5) (Fisher, Ref: BP231-100)
- Glacial Acetic acid (CAS 64-19-7) (Fisher, Ref: A38S-500)
- Dibutylamine (CAS 111-92-2) (Sigma Aldrich, Ref: 471232)
- Methanol, HPLC Grade (CAS 67-56-1) (Fisher, Ref: A452-1)
- Ammonia Solution 28-30% (CAS 1336-21-6) (MilliporeSigma Ref: 105423)
- Methanol, Optima Grade (CAS 67-56-1) (Fisher, Ref: A456-4)
- Water, Optima Grade (CAS 7732-18-5) (Fisher, Ref: W6500)

Benchtop Supplies:

- 1.5 mL Microcentrifuge tube with snap cap (Fisher, Ref: 05-408-129)
- Deep-well 96-well plate, polypropylene, round bottom, 1mL well (Costar, Ref: 3959)
- Sealing film for 96-well plate (Axygen, Ref: PCRSP)
- MilliQ Water Filtration System
- 2 mL Microcentrifuge Tubes with snap caps (Thomas Scientific, Ref: 1159V26)
- Heating Block (37°C) in Fume Hood
- Incubator set to 37°C with Shaker
- Allegra X-12R Centrifuge (Beckman Coulter)
- SpeedVac, Vacuum Concentrator
- Benchtop Centrifuge

Mass Spectrometry Supplies/Equipment:

- Pulled-point glass HPLC vial inserts, 250 µL (Agilent, Ref: 5183-2085)
- HPLC Vials, 2 mL (J.G. Finneran, Ref: 32009M-1232)
- HPLC Vial Cap, pre-slit septa (ThermoScientific, Ref: C5000-45B)
- Acquity UPLC Column BEH C18 1.7µm 2.1x100mm Column (Waters, Ref: 186002352)

- Acquity UPLC BEH C18 1.7µm 2.1x5mm Pre-Column (Waters, Ref: 186003975)
- Waters Xevo TQ-S Mass Spectrometer with Water's I-Class Acquity UPLC with flow-through needle injector

METHODOLOGY

Prepare the following solutions:

- 1% BSA in water: Dissolve 0.5 g BSA in 50 mL MilliQ water. Store at 4°C.
- 1 mg/mL I0S0 in water: Make 1 mg/mL stock by adding 1 mL MilliQ water to 1 mg of I0S0 powder. Store in Teflon-septum capped glass vials sealed with Parafilm at -20°C.
- 1 mg/mL I0S6 in water: Make 1 mg/mL stock by adding 1 mL MilliQ water to 1 mg of I0S6 powder. Store in Teflon-septum capped glass vials sealed with Parafilm at -20°C.
- 2.5 µg I0S0 and I0S6 Aliquots for Aniline labelling: Since only10 µg I0S0 and 10 µg of I0S6 NREs were received from Biomarin, these NREs were reconstituted in 100 µL water and then aliquoted into four 25 µL fractions in labeled 1.5 mL microcentrifuge tubes. The NRE solutions were dried via Speed Vac at room temperature and the aliquots were stored at -80°C until use.
- 1 mg/mL (2.49 mM) D0A0 in water: Make 1 mg/mL stock by adding 1 mL MilliQ water to 1 mg of D0A0 powder. Store in Teflon-septum capped glass vials sealed with Parafilm at -20°C.
- 1 mg/mL (2.17 mM) D0S0 in water: Make 1 mg/mL stock by adding 1 mL MilliQ water to 1 mg of D0S0 powder. Store in Teflon-septum capped glass vials sealed with Parafilm at -20°C.
- 50 µg D0A0, and D0S0 Aliquots for Aniline labelling: Prepare the 1 mg/mL internal standard stocks (D0A0 and D0S0) divide into 50 µL aliquots in labeled 1.5 mL microcentrifuge tubes. Dry down the solutions via Speed Vac at room temperature and store the aliquots at -80°C until use.
- 20 mM HEPES-HCl, 3.3 mM CaCl2, pH 7.0. Measure 142.8 mg HEPES and 11 mg of CaCl2 into a 50 mL falcon tube. Add 15 mL H2O and dissolve. Adjust pH with NaOH and HCl to 7.0. Bring volume to 30 mL.
- 100 mM DTT Solution. Dissolvµe 15 mg DTT per 1.00 mL of MilliQ Water.
- Heparinase I, II, and III stocks (2.5 IU/mL)*: Dissolve Heparinase I, II, and III (0.5 IU) in 0.2 mL of 1% BSA in water. This is a final concentration of 2.5 IU/mL. Aliquot into 10 µL fractions and store at -20°C to avoid multiple freeze-thaw cycles. More dilute solutions may be made if less than 20 samples will be run at a time.
- Heparinases Mixture (100 mIU/mL). Combine 20 µL of each Heparinase I, II, and III enzyme solution (2.5 IU/mL) and dilute to 500 µL with 1% BSA.
- 1 M Sodium Cyanoborohydride in DMSO:Acetic Acid (7:3, v/v): For ~50 samples, weigh 58.35 mg of sodium cyanoborohydride into a 4 mL glass vial and dissolve in 649.7 µL of DMSO and 278.8 µL Glacial Acetic Acid. Vortex the vial and allow to sit for 30 min, then vortex again. Allow the vial and contents to sit at room temperature for an additional 90 minutes (total time of 2h) to ensure sodium cyanoborohydride is completely dissolved.
- Mobile Phase A (MPA), 8mM Acetic Acid, 5 mM dibutylamine (DBA) in 100% water. To prepare 500 mL of MPA, add 228.8 µL glacial acetic acid and 421.3 µL DBA to 500 mL Optima grade water. Mix well.
- Mobile Phase B (MPB), 8mM Acetic Acid, 5 mM dibutylamine (DBA) in 30:70 water:methanol. To prepare 500 mL of MPB, add 228.8 µL glacial acetic acid and 421.3 µL DBA to 150 mL Optima grade water. Dilute with 350 mL of Optima grade methanol. Mix well.

  ** Enzymes seem to be stable with up to 3 freeze-thaw cycles, so make aliquots such that you can keep the number of cycles to 3, discarding after 3 cycles.*

Day 1 : Heparinase Digestion
Day 2 : Aniline Tagging
Day 3 : Dry on SpeedVac
Day 4 : LC-MS/MS injection

Digestion with Heparinases:
Punch one 3 mm DBS disk per patient and put the punches into a well of a 1 mL deep well 96-well plate (Costar Ref: 3959). Prepare the enzyme incubation mixture (for 50 samples) by combining 2.5 mL of HEPES buffer and 150 µL of 100 mM DTT solution, mix via vortexing. Then, add 500 µL of heparinases and mix gently with pipette. Add 63 µL of this solution to each well of the deep-well plate (50 µL HEPES, 3 µL DTT, 10 µL Heparinases). Cover the 96-well plate with sealing film and incubate at 37°C, shaking at 250 rpm, for 16 h.

Aniline Labelling of NREs and Internal Disaccharides by Reductive Amination:

After overnight incubation, quench each sample with 400 µL of cold methanol, pipetting up and down 20 times to mix, and incubate over ice for 10 minutes to precipitate proteins. Centrifuge the plate for 15 min at 3,000 *g*. Transfer 400 µL of each supernatant to a 1.5 mL microcentrifuge tube and dry via SpeedVac. Begin preparing sodium cyanoborohydride reagent in 7:3 DMSO:acetic acid (v/v) 2 h in advance of aniline reaction start time.

Calibrators, controls, and samples are tagged with [$^{12}C_6$] aniline. Internal Standards (ISTDs) are tagged with [$^{13}C_6$] aniline. Tagging must be performed under the hood. Layer the aniline with nitrogen and protect from light before storage. Set the heat block to 37°C (±2°C).

Add 15 µL of [$^{12}C_6$] aniline and 15 µL of 1M $NaCNBH_3$ to each dry heparinase-digested sample in a 2 mL microcentrifuge tube, then vortex the samples. Add 15 µL of [$^{13}C_6$] aniline and 15 µL of 1M $NaCNBH_3$ to one vial of each dry I0S0 and I0S6 NRE (2.5 µg) and to one vial of each dry D0S0 and D0A0 disaccharide (50 µg), then vortex the samples. Incubate samples and standards at 37°C for 30 minutes, then vortex again. Leave samples and standards to react at 37°C for an additional 15.5h (16h total) without shaking.

After 16h reacting, remove the tubes from the heat and dry via SpeedVac at room temperature for at least 24h.

Preparation for LC-MS/MS Analysis:
Reconstitute the dried [$^{13}C_6$] aniline labelled NRE ISTDs (I0S0 and I0S6) with 200 µL of Mobile Phase A to make 12.5 ng/µL and vortex. Reconstitute the dried [$^{13}C_6$] aniline labelled disaccharide ISTDs (D0S0 and D0A0) with 500 µL of Mobile Phase A to make 100 ng/µL solutions and vortex. Allow the dried ISTDs to incubate at room temperature for 30 minutes before vortexing again, then centrifuging for 15 min at 3,000 *g* to pellet particulates. Store the reconstituted ISTD solutions in labelled glass vials with Teflon-septum screw caps, sealed with Parafilm, at -20°C if not used immediately.

In order to reconstitute 50 dried [$^{12}C_6$] aniline labelled samples, prepare a 5 mL solution of 125 ng/mL I0S0 and I0S6 NRE ISTDs in MPA by combining 50 µL of I0S0 12.5 ng/µL stock with 50 µL of I0S6 12.5 ng/µL stock and diluting to 5 mL with MPA. D0A0 and D0S0 ISTDs could also be added to the 5 mL reconstitution solution if desired. Mix the 5 mL reconstitution solution well, then reconstitute dried [$^{12}C_6$] aniline labelled samples by adding 100 µL of the 125 ng/mL I0S0 and I0S6 in MPA stock. Vortex each sample and allow to incubate at room temperature for 30 minutes before vortexing a second time and centrifuging for 15 min at 3,000 *g*. Without disturbing the pellet, carefully transfer the liquid to a 2 mL HPLC vial containing a 250 µL pulled-point glass insert.

LC-MS/MS Analysis:

Aniline-labelled NREs and internal disaccharides were separated using an Acquity UPLC Column BEH C18 1.7µm 2.1x100mm Column (Waters, Ref: 186002352) fitted with an Acquity UPLC BEH C18 1.7µm 2.1x5mm Pre-Column (Waters, Ref: 186003975) connected to a Waters I-Class Aquity UPLC with a flow-through needle. The column was held at 40ºC. The markers were eluted in a 15-minute UPLC gradient program with 8mM acetic acid, 5 mM dibutylamine (DBA) in water (MPA) and 8mM acetic acid, 5 mM dibutylamine (DBA) in 30:70 water:methanol (v/v) (MPB). See the program in the table below. A strong needle wash of 100% acetonitrile, a purge wash of MPA, and a seal wash of 90/10 water/acetonitrile were used.

UPLC Solvent Gradient Program:

Time (min)	Flow (mL/min)	%MPA	%MPB
0.00	0.35	99.0	1.0
2.00	0.35	99.0	1.0
11.00	0.35	0.2	99.8
12.00	0.35	0.2	99.8
13.00	0.35	99.0	1.0
15.00	0.35	99.0	1.0

Disaccharides were detected with a Waters Xevo TQ-S mass spectrometer with an ESI source in negative mode. See mass spec parameters and MRMs below. Waters MassLynx Software and TargetLynx was used to analyze the data.

Xevo TQ-S Parameters

Parameter	Setting
Polarity	ESI-
Capillary (kV)	2.0
Source Temperature (ºC)	150
Desolvation Temperature (ºC)	600
Cone Gas Flow (L/hr)	150
Desolvation Gas Flow (L/hr)	900
Collision Gas Flow (mL/min)	ON
Collision Gas	Argon

Marker Names, Retention Times, MRMs, and Tuning Information for Xevo TQ-S

Analyte	Precursor	Product	Cone	Collision	
IoSo - [$^{12}C_6$]	510.94	430.81	36	20	
IoSo - [$^{13}C_6$]	516.55	436.63	34	23	
IoS6 - [$^{12}C_6$]*	591.07	431.01	44	32	
IoS6 - [$^{13}C_6$]*	597.03	436.77	50	34	
DoAo - [$^{12}C_6$]	455	157	35	20	MRM From ARUP Protocol
DoAo - [$^{13}C_6$]**	461	157	35	20	MRM From ARUP Protocol
DoSo - [$^{12}C_6$]	493	377	35	20	MRM From ARUP Protocol
DoSo - [$^{13}C_6$]**	499	383	35	20	MRM From ARUP Protocol

* IoS6 peak is can also be seen in the IoSo MRM channel. This is likely due to desulfation of the ion in the source. Due to high background and low IoS6 signal in the IoS6 MRM channel, the IoS6 peaks were processed through the IoSo MRM channel in order to achieve a more accurate peak integration. See example chromatograms.

** DoAo and DoSo - [$^{13}C_6$] ISTDs were not run with each assay because these markers can be seen using the Kubaski Method. DoAo and DoSo values were calculated relative to the IoSo - [$^{13}C_6$] ISTD peak for reference.

EXAMPLE CHROMATOGRAMS:

Below: IoS6 - [$^{12}C_6$] Peak at 591.07 > 431.01, showing some background interference for some peaks.

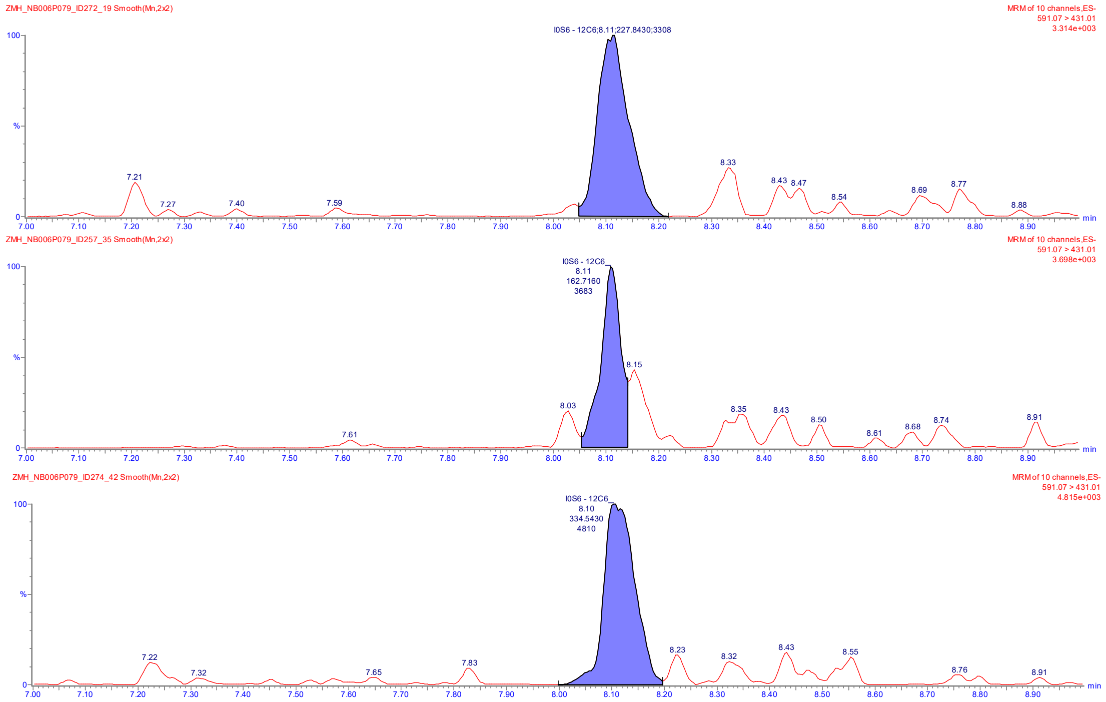

Marker: I0S0 MPS-I (ID: 106) Newborn DBS

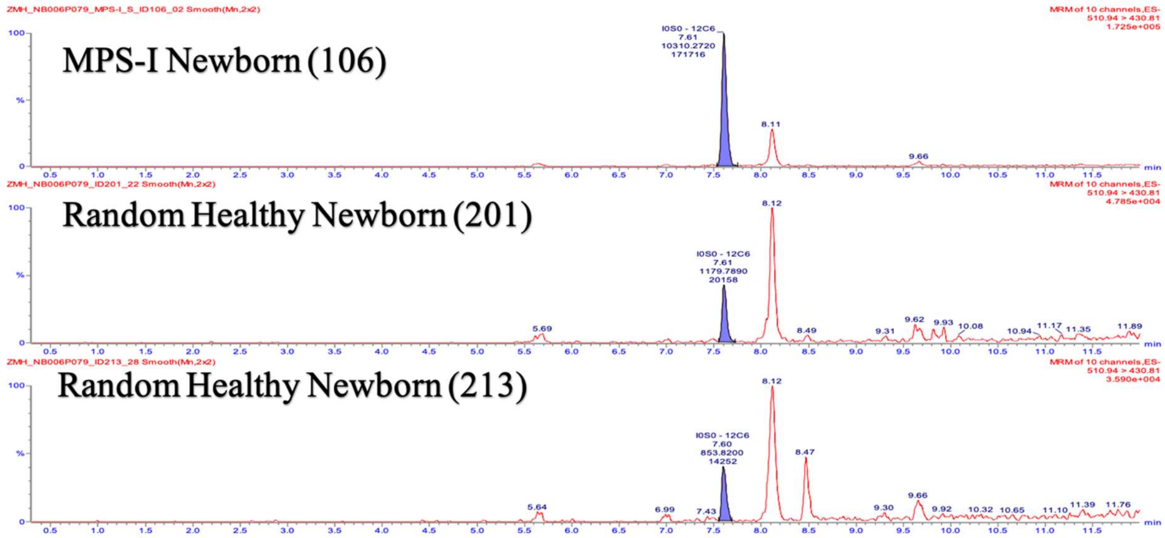

Supplementary Material: SensiPro Lite Method SOP

Marker: D0S0 MPS-I (ID: 106) Newborn DBS

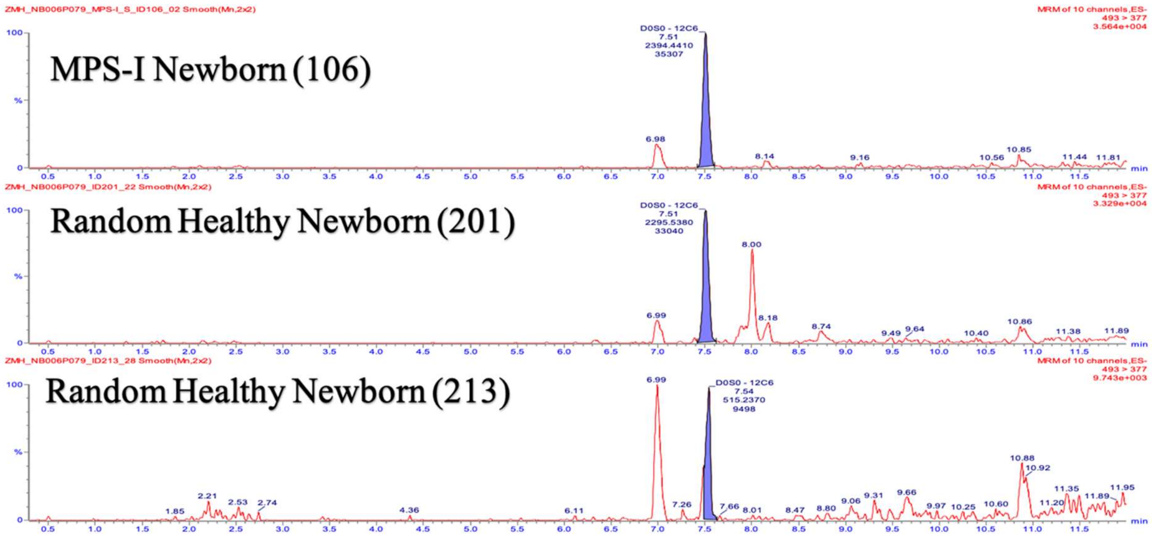

Marker: I0S6 (I0S0 trace) MPS-I (ID: 106) Newborn DBS

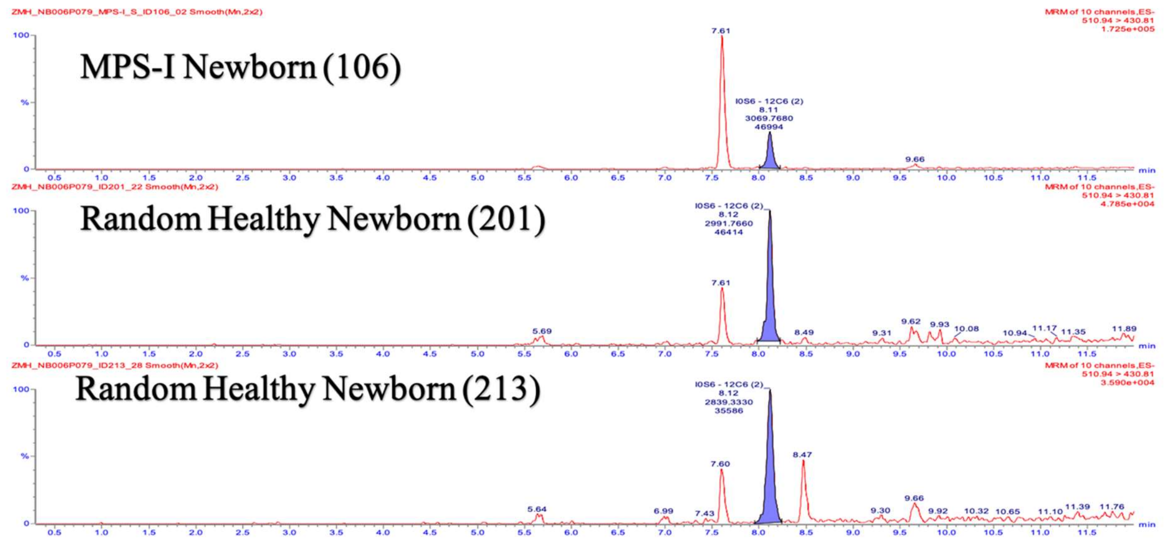

Supplementary Material: SensiPro Lite Method SOP

Marker: D0A0 MPS-I (ID: 106) Newborn DBS

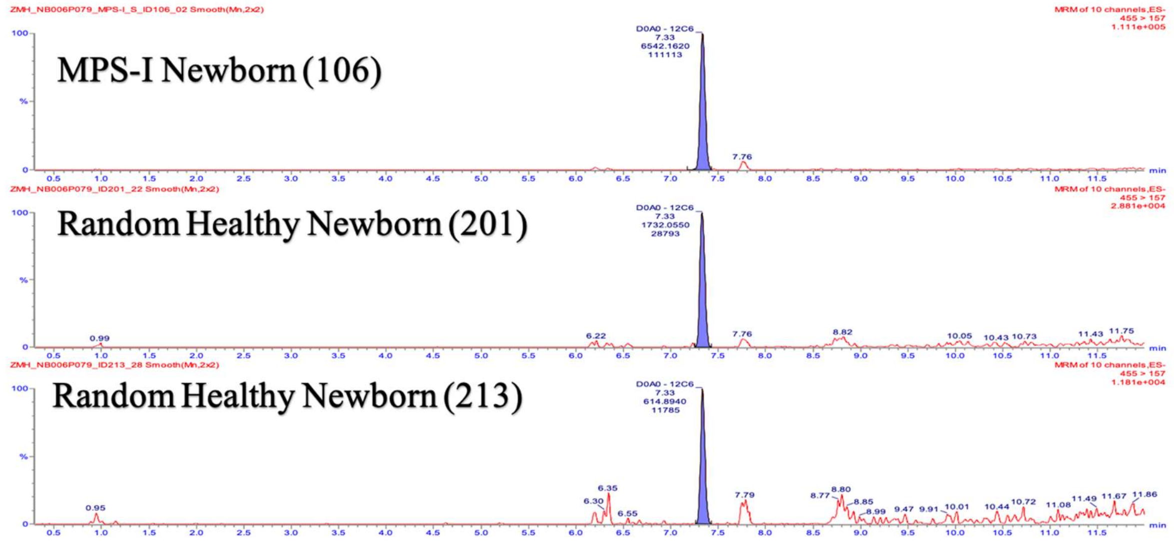

Supplement: Supplementary file 1 [file IJNS-06-00069-s001.zip › IJNS-06-00069-s001.pdf]
